# Supplementary material for: Palladium-Catalyzed Mizoroki–Heck and Copper-Free Sonogashira Coupling Reactions in Water Using Thermoresponsive Polymer Micelles
Source: Polymers (Basel). 2021 Aug 13;13(16):2717. doi: 10.3390/polym13162717 (PMC8402173; doi:10.3390/polym13162717)
Supplement: Supplementary file 1 [file polymers-13-02717-s001.zip › polymers-1310378-supplementary.pdf]

Supplementary Materials for

Palladium-Catalyzed Mizoroki-Heck and Copper-Free Sonogashira Coupling Reactions in Water Using Thermoresponsive Polymer Micelles

Noriyuki Suzuki[1,*], Shun Koyama[1], Rina Koike[1], Nozomu Ebara[1], Rikito Arai[1], Yuko Takeoka[1], Masahiro Rikukawa[1], Fu-Yu Tsai[2,*]

1) Department of Materials and Life Sciences, Faculty of Science and Technology, Sophia University, 7-1 Kioi-cho, Chiyoda-ku, Tokyo 102-8554, Japan
2) Institute of Organic and Polymeric Materials, National Taipei University of Technology, 1, Sec. 3, Chung-Hsiao E. Rd., Taipei 10608, Taiwan

1. Materials and Methods 3
1.1. General 3
1.2. Preparation of the homopolymer PNIPAAm. 4
1.3. Preparation of the copolymer Poly(NIPAAm-b-AMPSNa) ***NA-T****.* 4
1.4 Removal of the trithiocarbonate terminus in the PNIPAAm-b-PAMPSNa: ***NA***. [12] 4
1.5. Preparation of the copolymer of PDEAAm. 5
1.6. Preparation of the copolymer PDEAAm-b-PSSNa ***DS-T*** 5
1.7. Removal of trithiocarbonate terminus from ***DS-T****; synthesis of* ***DS****.* 5
1.8. Preparation of the diblock copolymer PDEAAm-b-PAMPSNa ***DA-T****.* 5
1.9. Removal of trithiocarbonate terminus from ***DA-T****; synthesis of* ***DA****.* 6
1.10. Mizoroki-Heck reactions in water using the copolymers, initial study. 6
1.11. Mizoroki-Heck reactions in water using the copolymers catalyzed by ***1****.* 6
1.12. Scanning transmission electronic microscopy (STEM) images after Mizoroki-Heck reactions in water using ***1****.* 8
1.13. Sonogashira coupling reactions in water using the copolymers. 9
1.14. Study on extraction efficiency3.6 Evaluation of extraction efficiencies 9
1.15. Calculation of E-factor in Mizoroki-Heck reaction. 9
1.16 Calculation of E-factor in Sonogashira reaction. 10
2. Spectroscopic Data 12
2.1. The homopolymer PNIPAAm. 12
2.2. The copolymer PNIPAAm-b-PAMPSNa with the RAFT fragment ***NA-T****.* 12
2.3. The copolymer PNIPAAm-b-PAMPSNa in which the trithiocarbonate terminus was removed: ***NA***. [12]13
2.4. The homopolymer PDEAAm. 13
2.5. The copolymer PDEAAm-b-PSSNa with S terminus ***DS-T*** 15
2.6. The copolymerPDEAAm-b-PSSNa without S terminus ***DS****.* 15

*2.7. The diblock copolymer PDEAAm-b-PAMPSNa with trithiocarbonate terminus **DA-T**.* 16
*2.8. The copolymer PDEAAm-b-PAMPSNa in which trithiocarbonate terminus was removed **DA**.* 16
*2.9 Heck product n-butyl cinnamate **5aa*** 17
*2.10. 2-Ethylhexyl (E)-cinnamate **5ab**.* 18
*2.11. Butyl (E)-3-(4-acetylphenyl)acrylate **5ca**.* 19
*2.12. Butyl (E)-3-(4-methoxyphenyl)acrylate **5da**.* 20
*2.13. N-Isopropyl-(E)-cinnamamide **5ac**.* 22
*2.14. (E)-Stilbene **5ad**.* 25
*2.15. (E)-1-Methyl-2-styrylbenzene **5ae**.* 26
*2.16. (E)-1-fluoro-4-styrylbenzene **5af**.* 27
*2.17. 1-Methoxy-4-(phenylethynyl)benzene 7**da*** 29
*2.18. 1-(4-(Phenylethynyl)phenyl)ethan-1-one 7**ca**.* 30
*2.19. 1-(Phenylethynyl)naphthalene 7**fa**.* 31
*2.20. 4-(Phenylethynyl)benzonitrile 7**ha**.* 32
*2.21. 2-(Phenylethynyl)pyridine 7**ia**.* 34
*2.22. tert-Butyl((4-methoxyphenyl)ethynyl)diemthylsilane 7**eb**.* 35
*2.23. 2-((tert-Butyldimethylsilyl)ethynyl)-6-methylpyridine 7**jb**.* 36
*2.24. 1-Methoxy-4-(oct-1-yn-1-yl)benzene 7**ec**.* 37
*2.25. (E)-But-1-en-3-yne-1,4-diyldibenzene 7**ka**.* 38
3. References 39

1. Materials and Methods

1.1. General

The preparation of copolymers was conducted under an argon atmosphere by using standard Schlenk techniques, unless otherwise mentioned. *N*-Isopropyl acrylamide (NIPAAm) was purchased from Kanto Chemical Co., Inc. and recrystallized from hexane/toluene prior to use. 2,2'-Azobis(isobutyronitrile) (AIBN) and dimethylacetamide (DMA) were purchased from Kanto Chemical Co., Inc. and used without further purification. Sodium dodecyl sulfate (SDS) and 4,4'-azobis(4-cyanovaleric acid) (V-501) were purchased from FUJIFILM Wako Pure Chemical Corporation and used as received. *N*,*N*-Diethylacrylamide was purchased from Tokyo Chemical Industry Co., Ltd. and distilled prior to use. Styrene was purchased from Tokyo Chemical Industry Co., Ltd., distilled and kept under argon. Sodium *p*-styrenesulfonate, 2-acrylamido-2-methylpropanesulfonic acid, dichlorobis(triphenylphosphine)palladium, 2,9-diphenyl-1,10-phenanthroline, Iodobenzene, *n*-butyl acrylate, diisopropylethylamine, tris[2-(dimethylamino)ethyl]amine and α-methylstyrene were purchased from Tokyo Chemical Industry Co., Ltd. and used as received. Other aryl halides, alkenes, ethynylarene and palladium catalysts were purchased and used as received. XPhos and Triton X-100 were purchased from Sigma-Aldrich Co. LLC. and used without further purification.

The palladium complex **1** was prepared from 2,9-diphenyl-1,10-phenanthroline and dichlorobis(acetonitrile)palladium according to the literature.[1] The RAFT agent **2a** was prepared according to the reported method in the literature. [2] The RAFT agent **2b** was prepared accoriding to the literature. [3] Triethylammonium hypophosphite was prepared from triethylamine and hypophosphinic acid in toluene. The diblock copolymer **NS** was prepared as previously reported. [4-11]

Dialysis was performed using Spectra/Por® RC tubing (MWCO: 3.5kD). Deionized water was obtained on WE-200 (Yamato Scientific Co., Ltd.). NMR spectra were recorded on JEOL ECA 500 and Bruker Avance III HD400 spectrometers. Gel permeation chromatography (GPC) was measured on HLC-8320 GPC (Tosoh Corporation) equipped with Shodex GPC LF-804 columns (Showa Denko K.K.) using *N*,*N*-dimethylformamide (DMF) (0.1 wt% LiBr) as eluent; the molecular weight of the polymers was determined based on monodispersed poly(ethylene oxide) as standard.

Dynamic light scattering (DLS) measurements were made with DLS-8000 and ELSZ-2000ZS (Otsuka Electronics Co., Ltd.). For DLS analysis, the aqueous solutions of copolymers (0.1 wt%) **NA** (m = 20, n = 8), **DS** (m = 23, n = 29), **DA** (m = 31, n = 4) were employed for DLS analysis. Scanning transmission electron microscopy (STEM) was recorded on S-8000 (Hitachi High-Tech

Corporation) Transmittance was recorded on Shimadzu UV-2550. Shear Viscosity was measured on a SVM3000 viscometer (Anton Paar) at 25 °C.

1.2. Preparation of the homopolymer PNIPAAm.

A thoroughly dried Schlenk tube (100 mL) was filled with argon. In this tube, RAFT agent **2b** (51 mg, 0.23 mmol), NIPAAm (0.54 g, 4.68 mmol), 2,2'-azobis(isobutyronitrile) (AIBN) (12 mg, 0.08 mmol) were dissolved in dimethylacetamide (DMA) (6 mL) and degassed in three freeze-pump-thaw cycles. The mixture was stirred at 60 °C for 24 h, and the reaction mixture was poured into hexane/diethyl ether (75/75 mL) to precipitate yellow solid. After the solvent was decanted, the yellow solid was dissolved in chloroform, the solution was collected and the solvent was removed in vacuo to leave the homopolymer PNIPAAm as yellow solid (363 mg, 62%). The molecular weight was determined by ^1H NMR spectroscopy. DP (degree of polymerization) = 30, M_n = 3,600 by ^1H NMR. ^1H NMR (D_2O, $Me_3SiCH_2)_3SO_3Na$, 500 MHz): δ 1.15 (CH_3), 1.6 (CH_2), 2.0-2.2 (C*H*), 3.89 (C*H*), 7.24-7.34 (br, P*h*).

1.3. Preparation of the copolymer Poly(NIPAAm-b-AMPSNa) ***NA-T****.*

The obtained PNIPAAm (227 mg, 0.063 mmol) was added to a dried Schlenk tube, and sodium 2-acrylamido-2-methylpropanesulfonic acid (0.19 g, 0.83 mmol), AIBN (6 mg, 0.04 mmol) were dissolved in DMSO (5 mL) in the tube. The mixture was degassed in three freeze-pump-thaw cycles. The tube was stirred at 65 °C for 17 h, and the yellow mixture was purified by dialysis for 3 days. The dialyzed yellow solution was dried in vacuo to afford the product polymer **NA-T** as white solid (380 mg, 89%). The molecular weight was determined by ^1H NMR spectroscopy. Our attempts to record GPC has been unsuccessful so far due to highly ionic property of the polymer. ^1H NMR (D_2O, $Me_3SiCH_2)_3SO_3Na$, 500 MHz): δ 1.15 (CH_3), 1.56 (CH_2), 2.0-2.2 (C*H*), 3.4-3.6 (br, SCH_2), 3.89 (C*H*), 7.24-7.34 (br, P*h*).

1.4 Removal of the trithiocarbonate terminus in the PNIPAAm-b-PAMPSNa: ***NA****.* [12]

The PNIPAAm-*b*-PAMPSNa **NA-T** (308 mg, 0.067 mmol), triethylammonium hypophosphite (82 mg, 0.4 mmol) and V-501 (11 mg, 0.04 mmol) were dissolved in DMSO (5 mL) and degassed by the freeze-pump-thaw method. The mixture was stirred at 80 °C for 3 h, and additional V-501 (11 mg, 0.04 mmol) was added to the solution. After the mixture was stirred at 60 °C for 17 h, the yellow solution was dialyzed. The resultant colorless solution with white precipitate was dried in vacuo to obtain the product as white solid (281 mg, 76%). The molecular weight was determined by ^1H NMR spectroscopy. DP of the PNIPAAm segment was 30, while PAMPSNa segment was 9, M_n = 5,700 by ^1H NMR. ^1H NMR (D_2O, $Me_3SiCH_2)_3SO_3Na$, 500 MHz): δ 1.15 (CH_3), 1.56 (CH_2), 2.0-2.2 (C*H*), 3.4-3.6 (br, SCH_2), 3.89 (C*H*), 7.24-7.34 (br, P*h*).

1.5. Preparation of the copolymer of PDEAAm.

In a dried Schlenk tube (25 mL), RAFT agent **2a** (92 mg, 0.36 mmol), *N*,*N*-diethylacrylamide (916 mg, 7.2 mmol) and AIBN (16 mg, 0.10 mmol) were dissolved in dimethylacetamide (7 mL). The mixture was degassed in three freeze-pump-thaw cycles, and was stirred at 60 °C for 24 h. The solution was poured into hexane (400 mL) and yellow precipitated was dissolved in chloroform. The volatiles were removed in vacuo to afford PDEAAm as yellow solid (529 mg, 53%). The average molecular weight of the polymer was determined as M_n = 2,300, M_w/M_n = 1.20 by gel permeation chromatography (GPC) analysis using poly(ethylene oxide) as standard, and M_n = 3,200 (*DP* = 23) by ^{1}H NMR. ^{1}H NMR (D_2O, $Me_3SiCH_2)_3SO_3Na$, 500 MHz): δ 1.10–1.25 (CH-CH_3), 1.5–1.8 (CH_2), 2.5–2.8 (C*H*), 3.2–3.5 (NCH_2), 7.24-7.34 (br, *Ph*).

1.6. Preparation of the copolymer PDEAAm-b-PSSNa ***DS-T***

In a thoroughly dried Schlenk tube, the obtained PDEAAm (255 mg, 0.08 mmol) was dissolved in DMSO (5 mL) and sodium p-styrene sulfonate (167 mg, 0.8 mmol) and AIBN (5.2 mg, 0.032 mmol) were added. The mixture was degassed in three freeze-pump-thaw cycles and stirred at 65 °C for 24 h. The yellow mixture was dialyzed for 2 days. The volatiles were removed from the dialyzed mixture in vacuo to afford the title compound as yellow solid (415 mg, 97%). ^{1}H NMR (D_2O, $Me_3SiCH_2)_3SO_3Na$, 500 MHz): δ 1.10–1.25 (CH-CH_3), 1.5–1.8 (CH_2), 2.5–2.8 (C*H*), 3.2–3.5 (NCH_2), 7.24-7.34 (br, *Ph*), 7.6–7.8 (C_6H_4).

1.7. Removal of trithiocarbonate terminus from ***DS-T****; synthesis of* ***DS****.*

In a thoroughly dried Schlenk tube (100 mL), the prepared **DS-T** (769 mg, 0.138 mmol), triethylammonium hypophosphite (0.172 g, 0.833 mmol) and V-501 (0.028g, 0.1 mmol) were dissolved in DMSO (8 mL). The mixture was degassed by three freeze-pump-thaw cycles, and heated at 80 °C for 24 h. The yellow solution was dialyzed for 25 h. The volatiles were removed in vacuo, to give the title compound as white solid (662 mg, 89%). DP was determined by ^{1}H NMR (m = 23, n = 9, M_n = 4,900). ^{1}H NMR (D_2O, $Me_3SiCH_2)_3SO_3Na$, 500 MHz): δ 1.10–1.25 (CH-CH_3), 1.5–1.8 (CH_2), 2.5–2.8 (C*H*), 3.2–3.5 (NCH_2), 7.24-7.34 (br, *Ph*), 7.6–7.8 (C_6H_4).

1.8. Preparation of the diblock copolymer PDEAAm-b-PAMPSNa ***DA-T****.*

The diethylacrylamide homopolymer was prepared as described above (*DP* = 34). The obtained PDEAAm (428 mg, 0.12 mmol), sodium 2-acrylamide-2-methylpropane sulfonate (APMSNa) 444 mg, 0.6 mmo) and AIBN (9 mg, 0.055 mmol) were dissolved in DMSO (7 mL) and degassed in three freeze-pump-thaw cycles. The mixture was stirred at 65 °C for 17 h. The yellow mixture was dialyzed, and the volatiles were removed in vacuo. The title compound was obtained as white solid (380 mg, 78%). ^{1}H NMR (D_2O, $Me_3SiCH_2)_3SO_3Na$, 500 MHz): δ 1.10–1.25 (CH-CH_3), 1.5 (CH_3), 1.6–1.8 (CH_2), 2.5–2.8 (C*H*), 3.2–3.5 (NCH_2 + SCH_2), 7.24-7.34 (br, *Ph*).

*1.9. Removal of trithiocarbonate terminus from **DA-T**; synthesis of **DA**.*

The obtained polymer **DA-T** (444 mg, 0.12 mmol) was dissolved in DMSO (6 mL), and triethylammonium hypophosphite (112 mg, 0.54 mmol) and V-501 (15 mg, 0.054 mmol) were added to this solution. The mixture was degassed by the freeze-pump-thaw method, and stirred at 80 °C for 3 h. Additional V-501 (15 mg, 0.054 mmol) was added to the solution. After the mixture was stirred at 60 °C for 17 h, the yellow solution was dialyzed. The resultant colorless solution with white precipitate was dried in vacuo to afford the product as white solid (281 mg, 76%). DP was determined by ^{1}H NMR (m = 34, n = 3, M_n = 5,100).

^{1}H NMR (D_2O, $Me_3SiCH_2)_3SO_3Na$, 500 MHz): δ 1.10–1.25 (CH-CH_3), 1.5 (CH_3), 1.6–1.8 (CH_2), 2.5–2.8 (C*H*), 3.2–3.5 (NCH_2 + SCH_2), 7.24-7.34 (br, *Ph*).

1.10. Mizoroki-Heck reactions in water using the copolymers, initial study.

Typical procedure for Mizoroki-Heck reactions in water using the thermo-responsive micelles is as follows. In a test tube with a screw cap, the copolymer (40 mg) was dissolved in deionized water (4 mL) and the solution was stirred. To this solution iodobenzene (102 mg, 0.5 mmol), *n*-butyl acrylate (128 mg, 1.0 mmol), $PdCl_2(PPh_3)_2$ (7.0 mg, 0.01 mmol) and diisopropylethylamine (129 mg, 1.0 mmol) were added and the mixture was stirred at 70 °C for 48 h. The grayish turbid suspension was cooled in an ice bath, and then diethyl ether (3 mL) was added and stirred for 1 h. The organic layer was taken up and extracted again with diethyl ether (3 mL) until the product was not detected by thin layer chromatograph. The extract was analyzed by gas chromatography using tetradecane as an internal standard to determine GC yield.

*1.11. Mizoroki-Heck reactions in water using the copolymers catalyzed by **1**.*

Typically, in a test tube with a screw cap, the copolymer (20 mg) was dissolved in deionized water (1 mL) and the solution was stirred. To this solution iodobenzene (102 mg, 0.5 mmol), *n*-butyl acrylate (128 mg, 1.0 mmol), tri-*n*-butylamine (185 mg, 1.0 mmol) were added and the mixture was stirred. Meanwhile the palladium complex **1** (2.4 mg, 0.005 mmol) was dissolved in *N*-methyl-2-pyrrolidone (400 μL) in a vial. To this yellow solution was added hydrazine monohydrate (1.6 mg, 0.032 mmol) and stirred for 10 seconds. This 40 μL of this solution was added to the test tube, and the mixture was stirred at 70 °C for 48 h. The turbid suspension was cooled in an ice bath, and then ethyl acetate (0.4 mL) was added and vigorously stirred, and centrifuged at 1000 rpm for 10 min (180 ×g). The organic layer was taken up and extracted again with ethyl acetate (0.4 mL) until the product was not detected by thin layer chromatograph. The extract was analyzed by gas chromatography using tetradecane as an internal standard to determine GC yield (97%).

Other reductants such as hydroquinone and ascorbic acid were also tried. These were added to the mixture that contains all compounds and the Pd catalyst. Table S1 includes the data that are not

shown in the body text (entries 6-8).

Table S1. Mizoroki-Heck reaction in water by **1**[a]

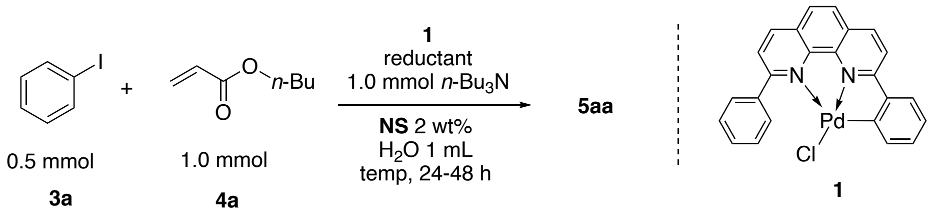

Entry	**1**/mol%	reductant/mol%	Temp/°C	Time/h	Yield/%	TON
1	0.5	none/ 0	100	48	46	92
2	0.5	H_2NNH_2/ 6.5	100	48	97	194
3	0.1	H_2NNH_2/ 0.65	100	24	99	990
4	0.01	H_2NNH_2/ 0.065	100	48	64	6,400
5[b]	0.01	H_2NNH_2/ 0.065	100	48	78	7,800
6[b]	0.1	H_2NNH_2/ 10	r.t.	24	0	-
7	0.1	Hydroquinone/ 10	100	24	65	650
8	0.1	L-Ascorbic acid/ 10	100	24	81	810

a) Conditions: iodobenzene (0.5 mmol), *n*-butyl acrylate (1.0 mmol), tri-*n*-butylamine (1.0 mmol), **NS** (2.0 wt% in water), H_2O (1 mL), 100 °C.

b) Tri-*n*-butylamine (0.6 mmol).

*1.12. Scanning transmission electronic microscopy (STEM) images after Mizoroki-Heck reactions in water using **1**.*

After the Mizoroki-Heck reaction, the extract was analyzed by gas chromatography using tetradecane as an internal standard to determine GC yield (97%). The remained aqueous layer was observed by STEM (Figure S1, a). To this aqueous solution was added the substrates and base and the second reaction was carried out. The aqueous layer after three cycles of reactions was also observed by STEM (Figure S1, b).

(a) after single reaction (b) after three times of reactions.

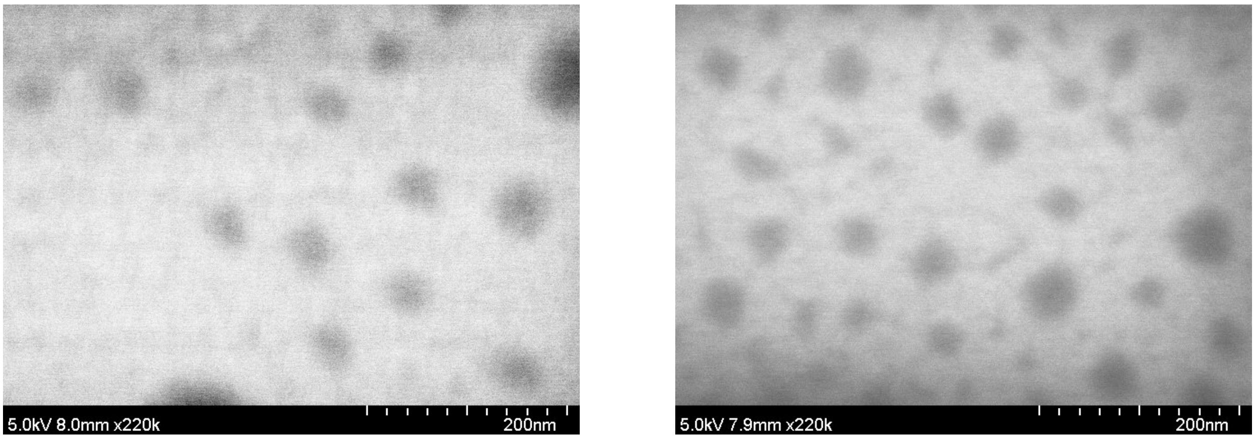

Figure S1. STEM images of the aqueous solutions after the Mizoroki-Heck reactions.

1.13. Sonogashira coupling reactions in water using the copolymers.

Typical procedure for Sonogashira coupling in water is as follows. In a dried test tube with a screw cap, copolymer **NS** (20 mg) was dissolved in deionized water (2 mL). To this solution were added 4-iodoanisole (117 mg, 0.5 mmol), ethynylbenzene (77 mg, 0.75 mmol) and $PdCl_2(PPh_3)_2$ (7.0 mg, 0.01 mmol) and triethylamine (110 mg, 1.0 mmol), the mixture was stirred at 70 °C for 24 h. The brown turbid solution was cooled in an ice-bath with stirring until the supernatant became clear. The precipitated brown solid was collected by filtration when possible. The product was characterized by ^1H NMR in $CDCl_3$. Otherwise, the solid was extracted with ethyl acetate and purified by column chromatograph on silica gel (hexane/ethyl acetate = 4/1).

1.14. Study on extraction efficiency3.6 Evaluation of extraction efficiencies

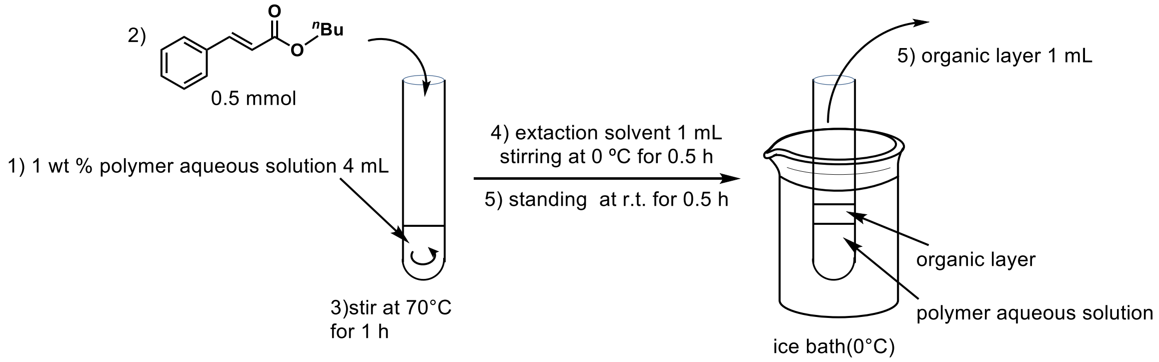

Efficiency of extraction process was estimated as follows.

Typically, in a test tube with screw cap, the block copolymers (42 mg) was dissolved in deionized water (4 mL) and the solution was stirred for 50 min. at rt. To this solution, *n*-butyl cinnamate (**5aa,** 102 mg, 0.5 mmol) was added and the mixture was stirred at 70 °C for 1 h. This mixture was cooled at 0 °C and ethyl acetate (1 mL) was added. The mixture was shaken at 120 rpm in a shaking apparatus at 0 °C for 0.5 h. The mixture was allowed to stand still for 0.5 h at ambient temperature, and then the organic layer was taken up and analyzed by gas chromatograph using tetradecane as an internal standard.

1.15. Calculation of E-factor in Mizoroki-Heck reaction.

In a test tube with a screw cap, **DA** (20 mg) was dissolved in purified water (2 mL) and the solution was stirred for 30 min at rt. And then, iodobenzene (102 mg, 0.5 mmol), *n*-butyl acrylate (96 mg, 0.75 mmol), $PdCl_2(PPh_3)_2$ (7.0 mg, 0.01 mmol) and diisopropylethylamine (97 mg, 0.75 mmol) were added and the mixture was stirred at 70 °C for 48 h. The turbid solution was cooled to rt and ethyl acetate (0.5 mL) was added and stirred for 1 h with a magnetic stirrer with 120 rpm using a stir bar (5 mmφ × 10 mm). The mixture was centrifuged for 5 minutes with 1000 rpm (180 ×g), the organic layer was taken up. The product **5aa** was obtained in 68% yield (94% GC

yield).

Wastes: 4-iodobenzene 6.1 mg, *n*-butyl acrylate 35.8 mg, diisopropylethylamine 97 mg, palladium catalyst 7 mg, copolymer 20 mg, ethyl acetate 450 mg, water as a reaction medium 2000 mg.

Product: 69.5 mg (68%)

E-factor = (6.1+35.9+97+7+20+450)/69.5 = 616.0/69.5 = 8.9 when water is not accounted for.

= (6.1+35.8+97+7+20+450+2000)/69.5 = 38 when water is included in waste.

1.16 Calculation of E-factor in Sonogashira reaction.

In a test tube with a screw cap, **NS** (20 mg) was dissolved in purified water (2 mL) and the solution was stirred for 30 min at rt. And then, 4-iodoanisole (117 mg, 0.5 mmol), ethynylbenzene (77 mg, 0.75 mmol), $PdCl_2(PPh_3)_2$ (7.0 mg, 0.01 mmol) and triethylamine (76 mg, 0.75 mmol) were added and the mixture was stirred at 70 °C for 24 h. The brown turbid solution was cooled to rt with stirring until the supernatant became clear. The precipitated brown solid was collected by filtration. The solid was characterized by ^{1}H NMR in $CDCl_3$.

Wastes: 4-iodoanisole 18.7 mg, ethynylbenzene 33.7 mg, triethylamine 75.9 mg, palladium catalyst 7 mg, copolymer 20 mg, water as a reaction medium 2000 mg, water for wash 3000 mg.

Product: 87.5 mg (84%)

E-factor = (18.7+33.7+75.9+7+20)/87.5 = 1.77 when water is not accounted for.

= (18.7+33.7+75.9+7+20+2000+3000)/87.5 = 58.9 when water is involved in waste.

^{1}H NMR of the precipitated product, washed with water.

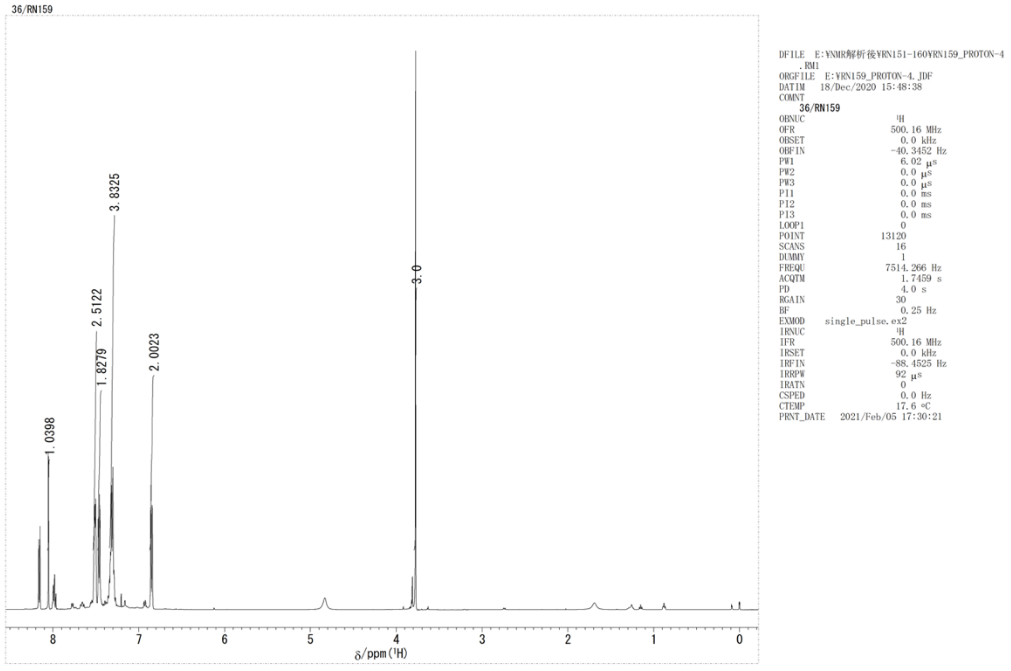

^{1}H NMR of the water layer in Sonogashira coupling

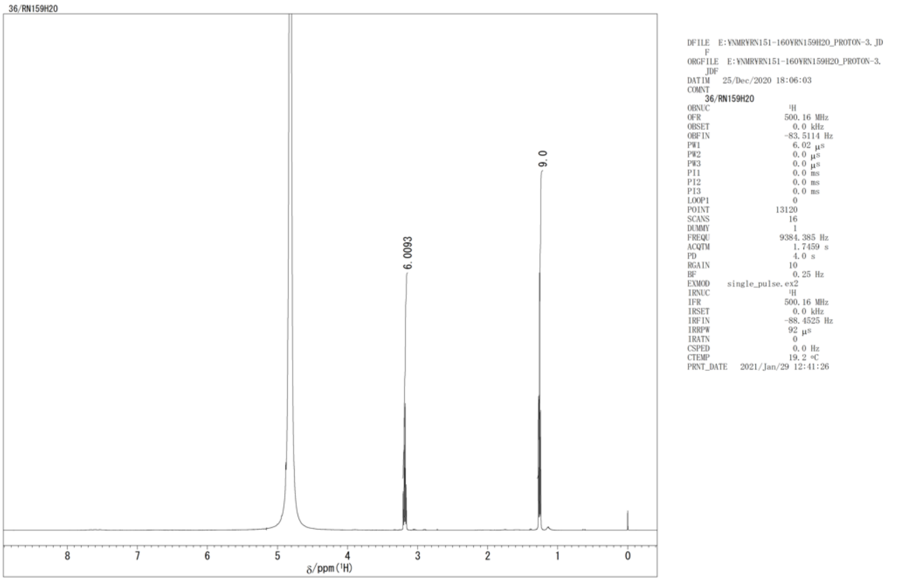
36/RN159H20
9.0
6.0093
δ/ppm (¹H)
8 7 6 5 4 3 2 1 0
DFILE E:¥NMR¥RN151-160¥RN159H20_PROTON-3.JDF
ORGFILE E:¥NMR¥RN151-160¥RN159H20_PROTON-3.JDF
DATIM 25/Dec/2020 18:06:03
COMNT 36/RN159H20
OBNUC ¹H
OFR 500.16 MHz
OBSET 0.0 kHz
OBFIN -83.5114 Hz
PW1 6.02 μs
PW2 0.0 μs
PW3 0.0 μs
PI1 0.0 ms
PI2 0.0 ms
PI3 0.0 ms
LOOP1 0
POINT 13120
SCANS 16
DUMMY 1
FREQU 9384.385 Hz
ACQTM 1.7459 s
PD 4.0 s
RGAIN 10
BF 0.25 Hz
EXMOD single_pulse.ex2
IRNUC ¹H
IFR 500.16 MHz
IRSET 0.0 kHz
IRFIN -88.4525 Hz
IRRPW 92 μs
IRATN 0
CSPED 0.0 Hz
CTEMP 19.2 °C
PRNT_DATE 2021/Jan/29 12:41:26

(Enlarged)

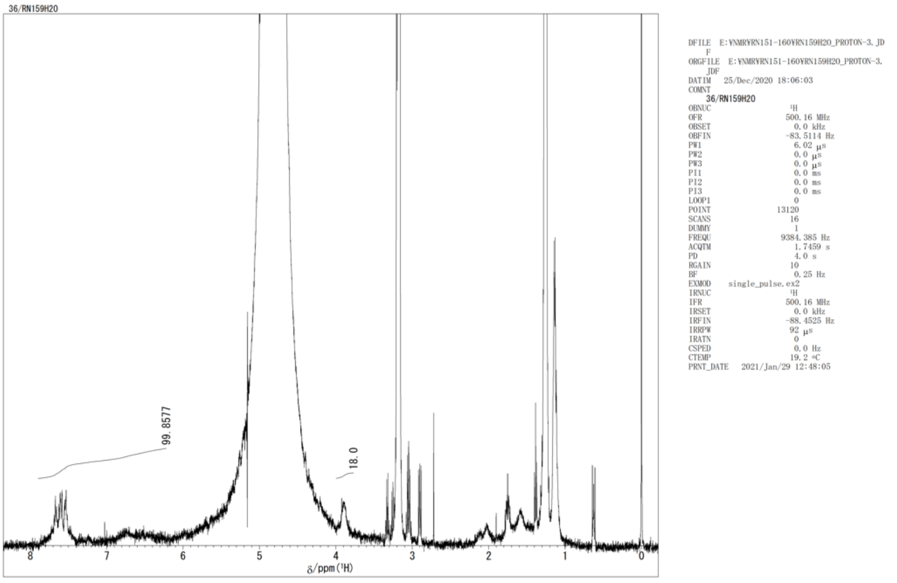
36/RN159H20
99.8577
18.0
δ/ppm (¹H)
8 7 6 5 4 3 2 1 0
DFILE E:¥NMR¥RN151-160¥RN159H20_PROTON-3.JDF
ORGFILE E:¥NMR¥RN151-160¥RN159H20_PROTON-3.JDF
DATIM 25/Dec/2020 18:06:03
COMNT 36/RN159H20
OBNUC ¹H
OFR 500.16 MHz
OBSET 0.0 kHz
OBFIN -83.5114 Hz
PW1 6.02 μs
PW2 0.0 μs
PW3 0.0 μs
PI1 0.0 ms
PI2 0.0 ms
PI3 0.0 ms
LOOP1 0
POINT 13120
SCANS 16
DUMMY 1
FREQU 9384.385 Hz
ACQTM 1.7459 s
PD 4.0 s
RGAIN 10
BF 0.25 Hz
EXMOD single_pulse.ex2
IRNUC ¹H
IFR 500.16 MHz
IRSET 0.0 kHz
IRFIN -88.4525 Hz
IRRPW 92 μs
IRATN 0
CSPED 0.0 Hz
CTEMP 19.2 °C
PRNT_DATE 2021/Jan/29 12:48:05

2. Spectroscopic Data

2.1. The homopolymer PNIPAAm.

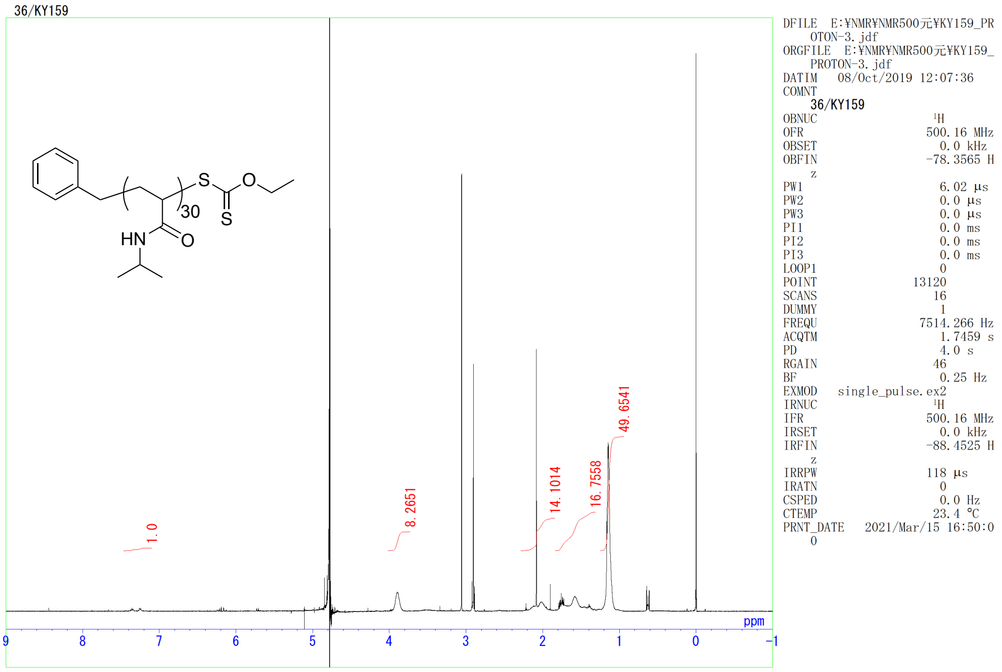

*2.2. The copolymer PNIPAAm-b-PAMPSNa with the RAFT fragment **NA-T**.*

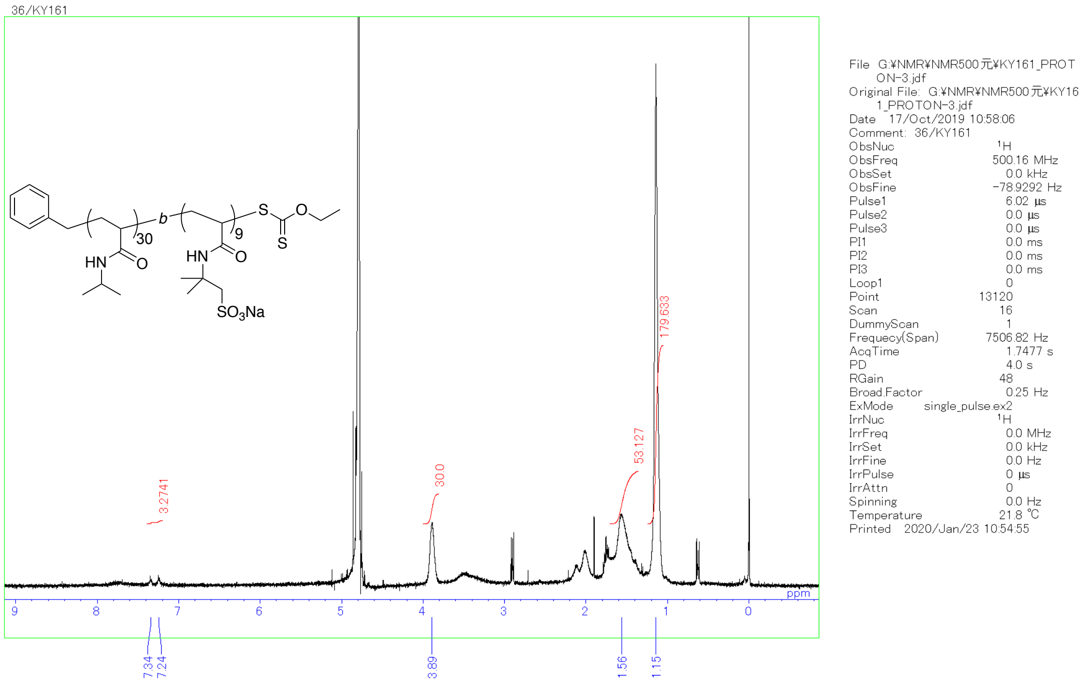

2.3. The copolymer PNIPAAm-b-PAMPSNa in which the trithiocarbonate terminus was removed: ***NA***. [12]

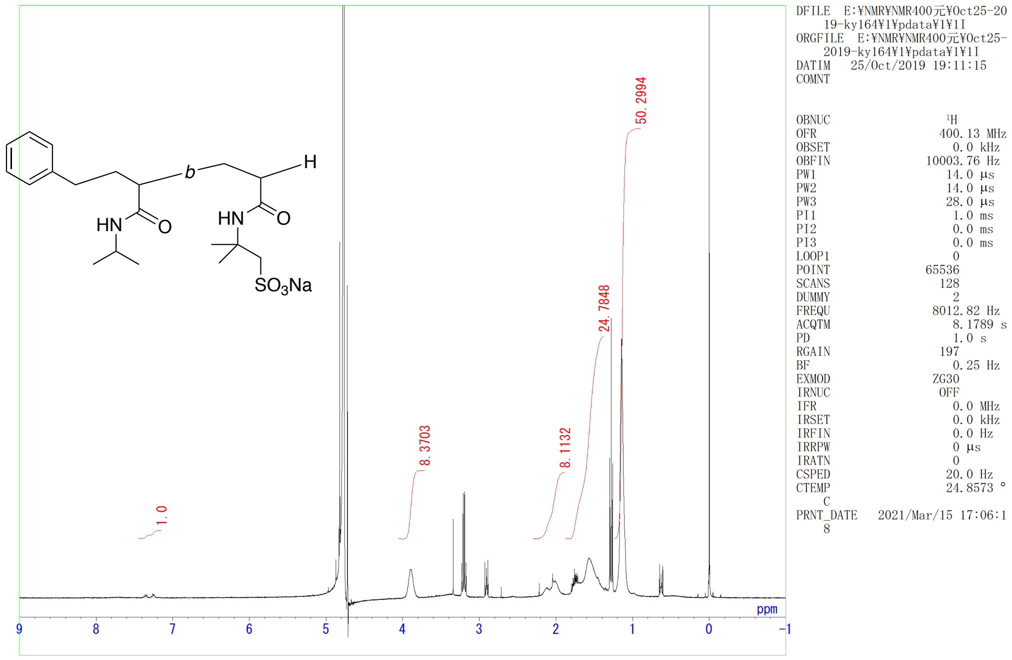

2.4. The homopolymer PDEAAm.

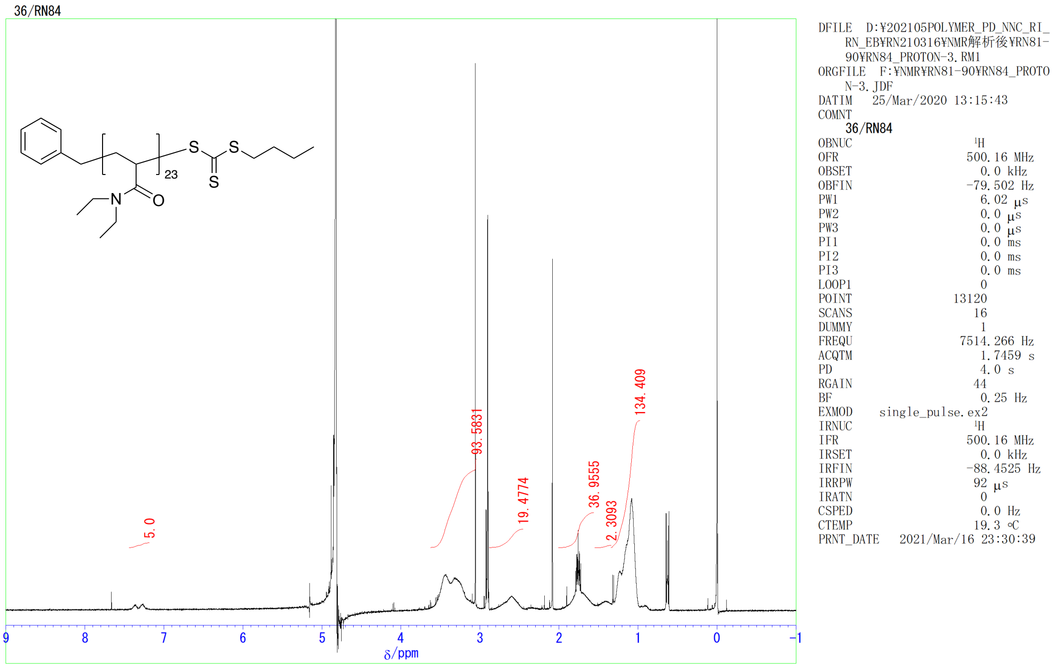

20200831_0831 EBcal200831_005 2021/01/26 16:33:45

クロマトグラム

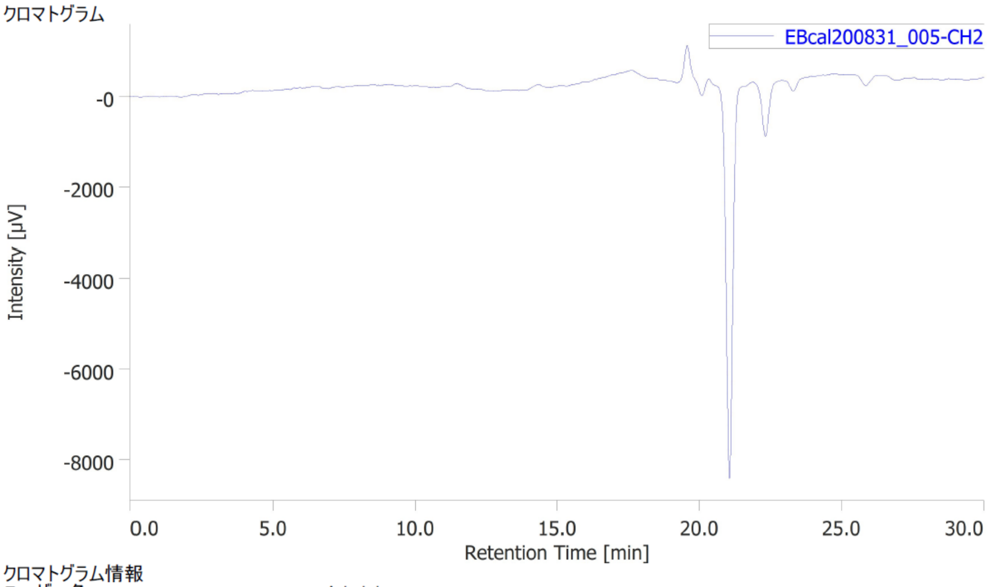

クロマトグラム情報
ユーザー名　Administrator
更新日時　2020/09/01 16:48:11
コメント
HPLC システム名　ChromNAV Lite
測定日　2020/09/01 13:03:15
注入量　10.0 [μL]
サンプル#　–
プロジェクト名　KY
取込時間　0.0 [min]
測定シーケンス
コントロールメソッド
ピークIDテーブル
検量線テーブル
追加情報
カラム情報
クロマトグラムラベル
測定済みシーケンス　20200831_0831 2020/08/31 14:32:46
クロマトグラム名　EBcal200831_005
サンプル名　RI-RN86
分子量分布曲線
分子量分布曲線 全区間

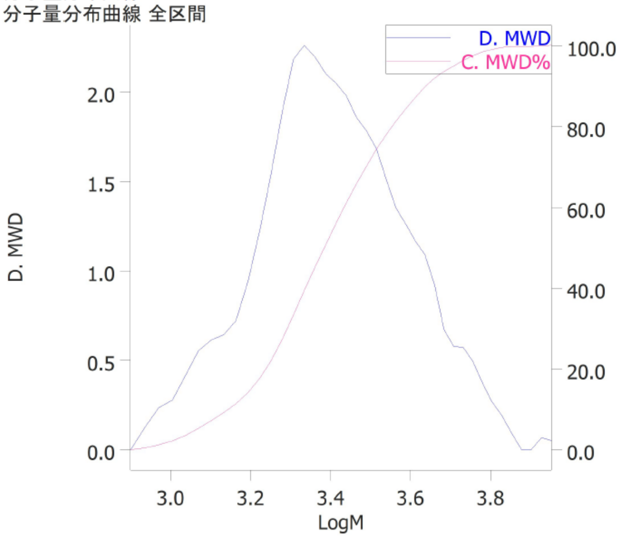

分子量計算結果
＜ファイル情報＞
ファイル名　20200831EB
ユーザー名　Administrator
更新日時　2020/09/01 16:38:50
コメント
＜分子量計算結果(全区間)＞

#	区間範囲	CH	Mp	Mn	Mw	Mz	Mv	Mw/Mn
1	全区間	2	----	2305	2771	3321	2771	1.2023

#	Mz/Mw	区間値	百分率	警告	サンプル名
1	1.1984	26550	----	外挿	RI-RN86

#	サンプルコメント
1	

＜分子量計算結果(区間別)＞

#	区間範囲	CH	ベースライン範囲	tR	Mp
1	15.0000 – 19.0000	2	15.0000 – 19.0000	17.6083	2079

#	Mn	Mw	Mz	Mv	Mw/Mn	Mz/Mw	区間値
1	2305	2771	3321	2771	1.2023	1.1984	26550

#	百分率	警告	サンプル名	サンプルコメント
1	100.00	外挿	RI-RN86	

GPC spectrum; Molecular weight distribution of PDEAAm.

2.5. *The copolymer PDEAAm-b-PSSNa with S terminus* ***DS-T***

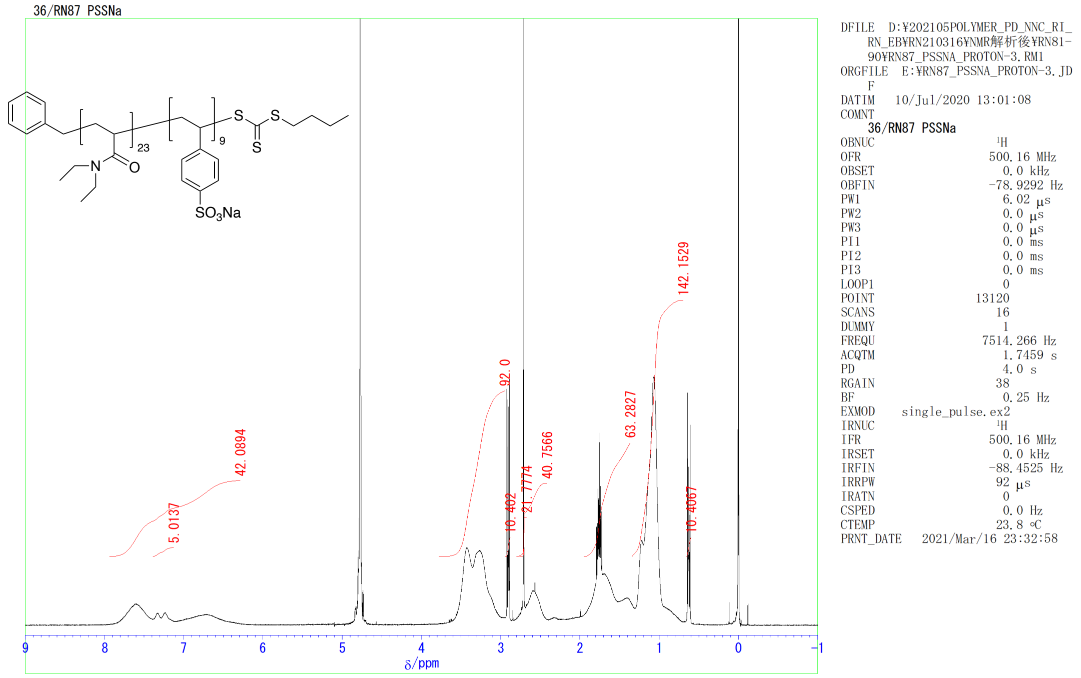

2.6. *The copolymerPDEAAm-b-PSSNa without S terminus* ***DS***.

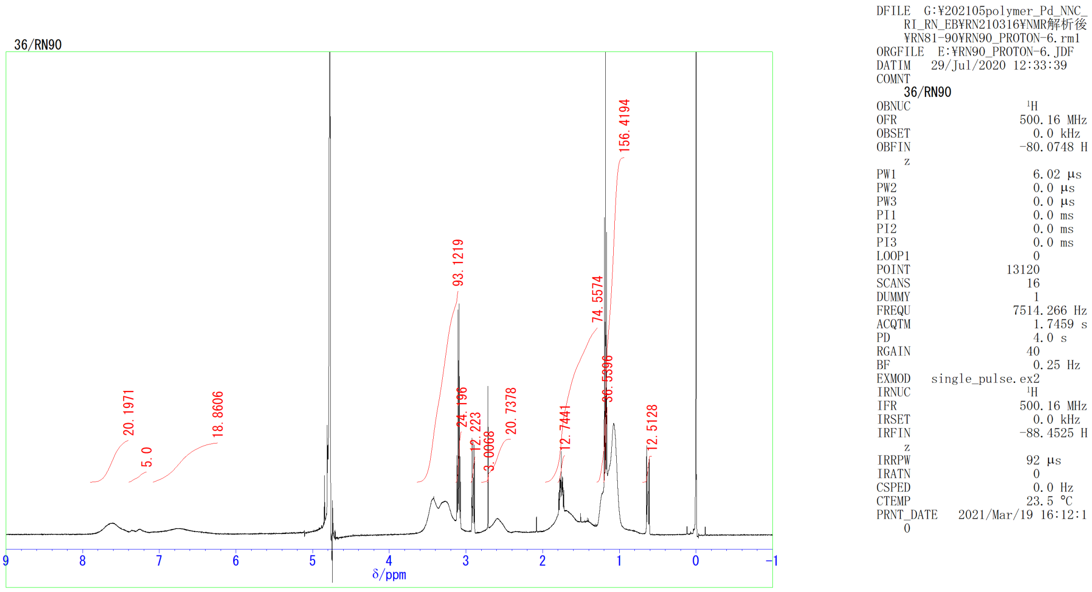

*2.7. The diblock copolymer PDEAAm-b-PAMPSNa with trithiocarbonate terminus **DA-T**.*

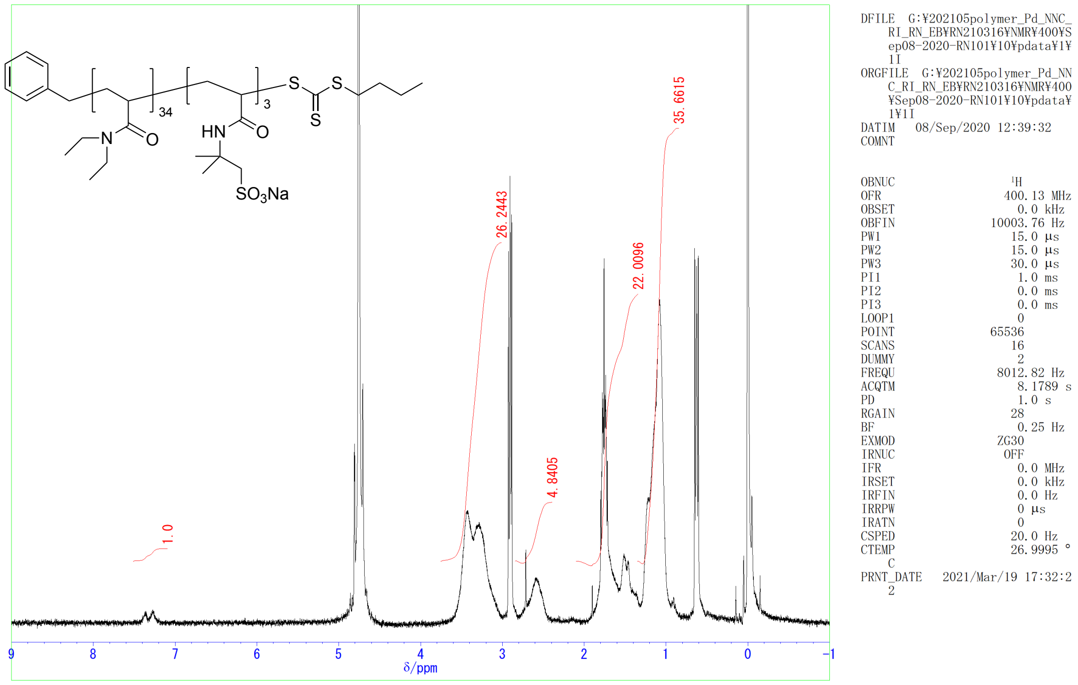

*2.8. The copolymer PDEAAm-b-PAMPSNa in which trithiocarbonate terminus was removed **DA**.*

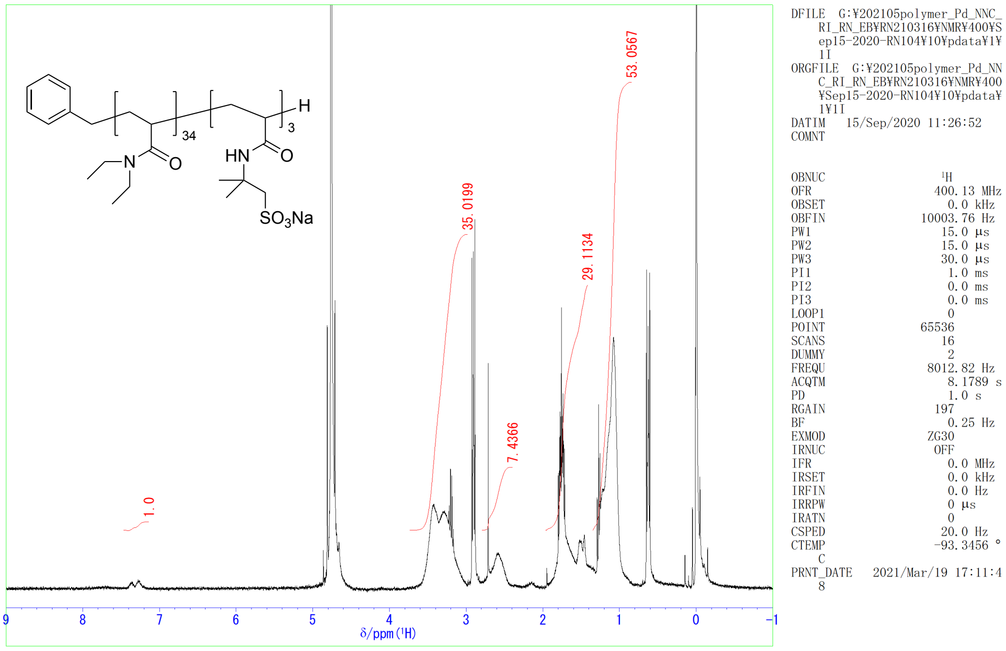

2.9 Heck product n-butyl cinnamate **5aa**

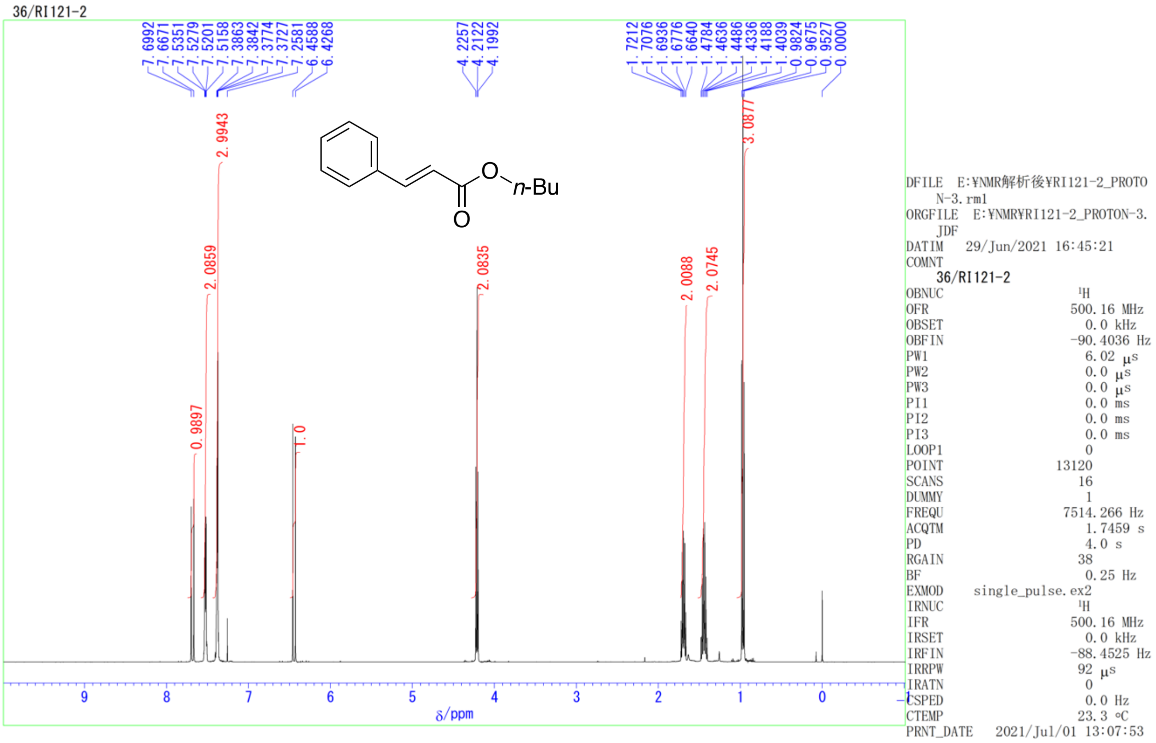

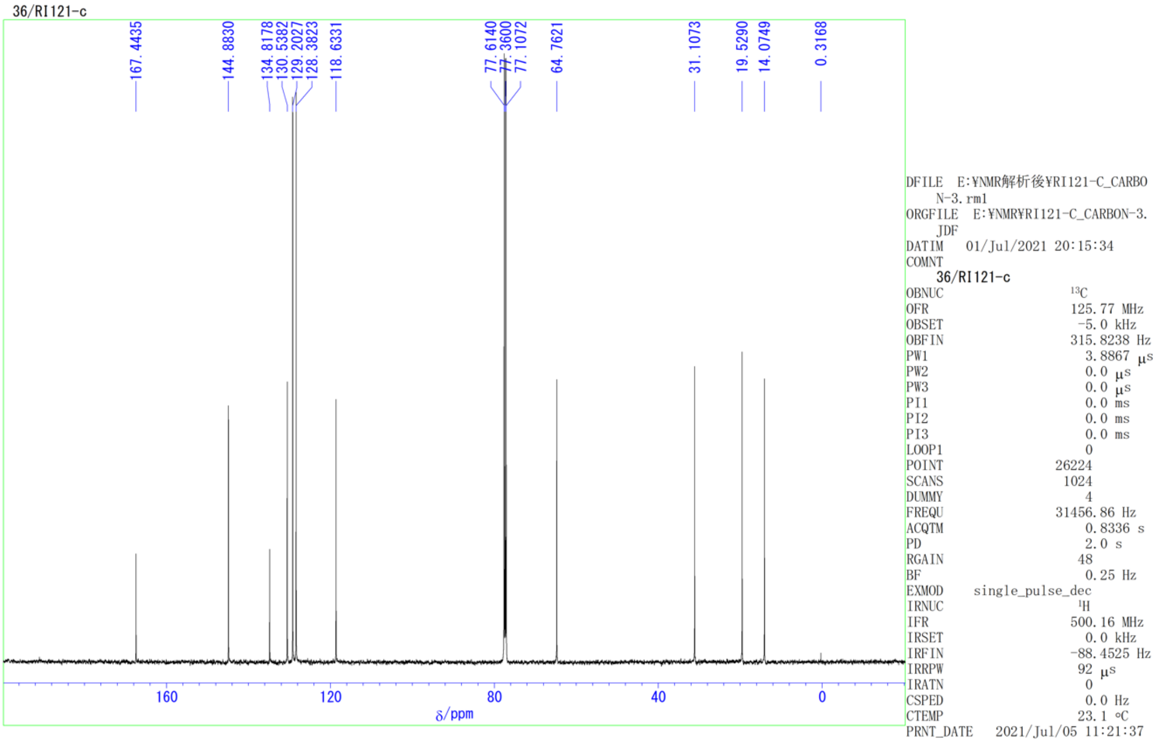

*2.10. 2-Ethylhexyl (E)-cinnamate **5ab**.*

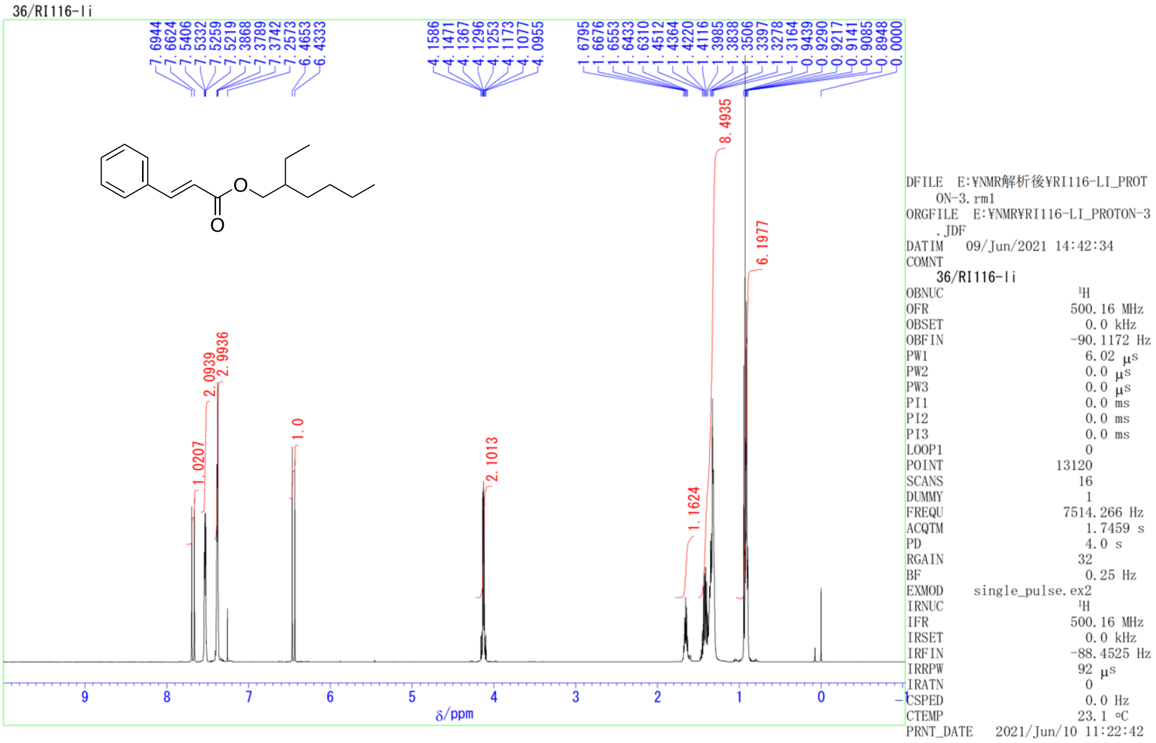

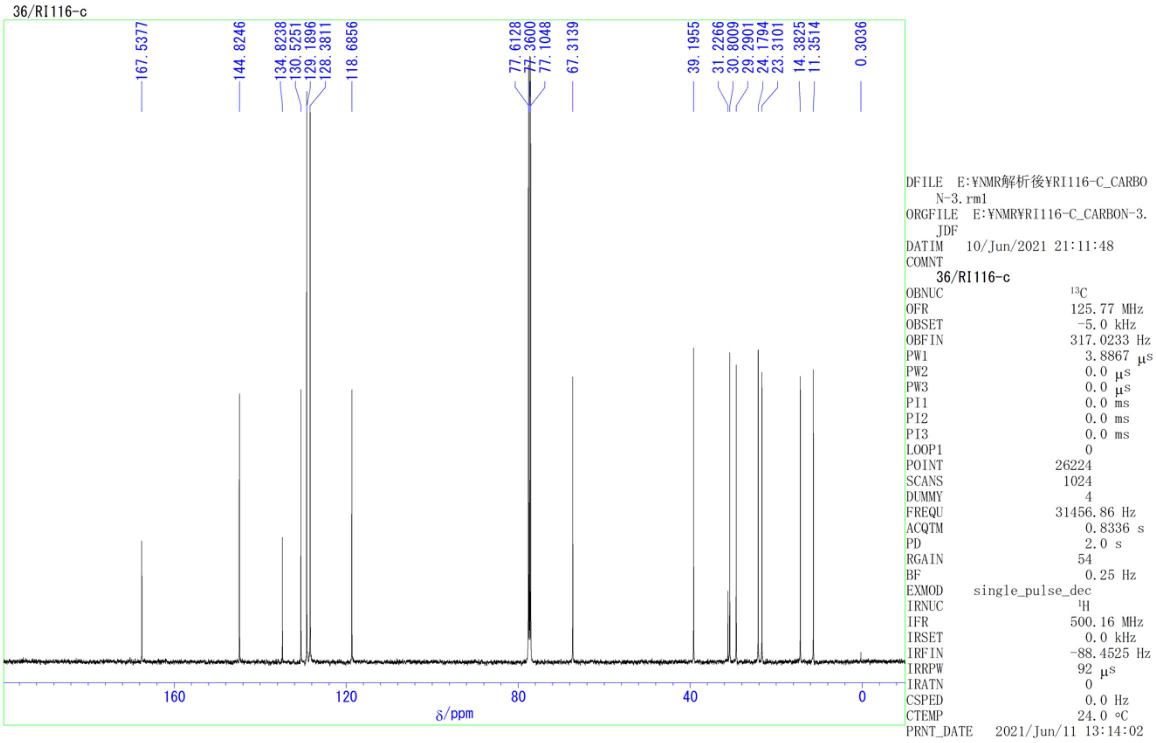

2.11. Butyl (E)-3-(4-acetylphenyl)acrylate ***5ca****.*

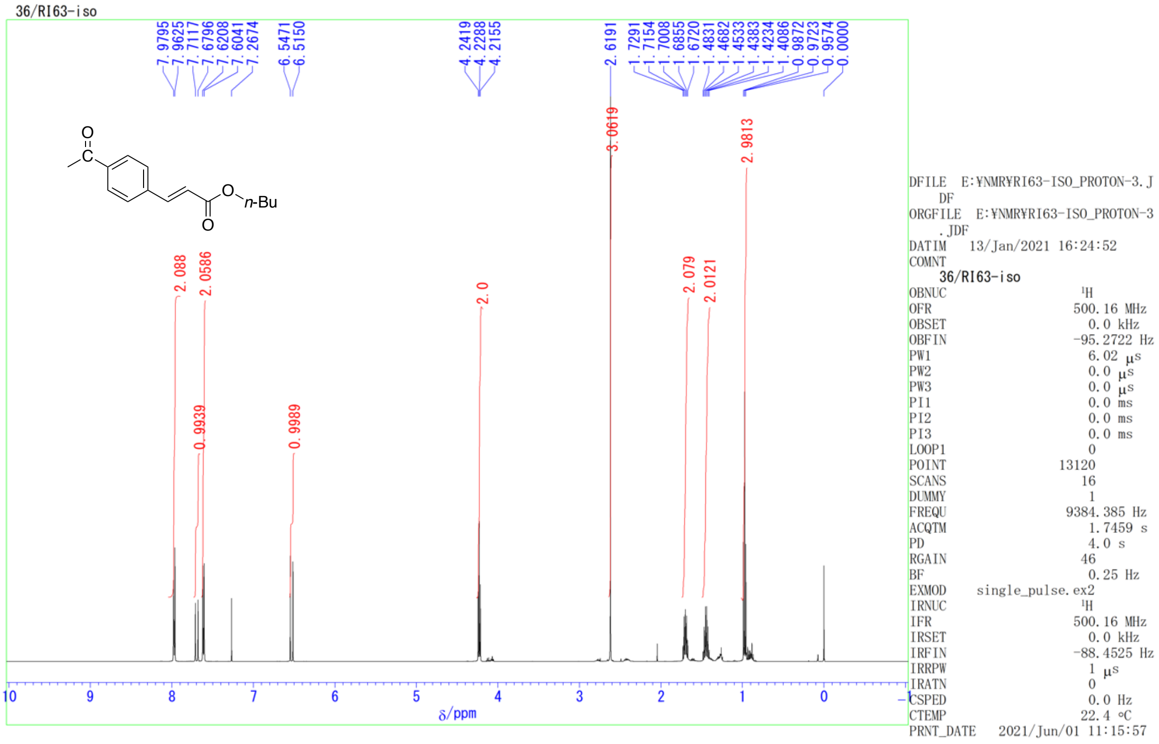

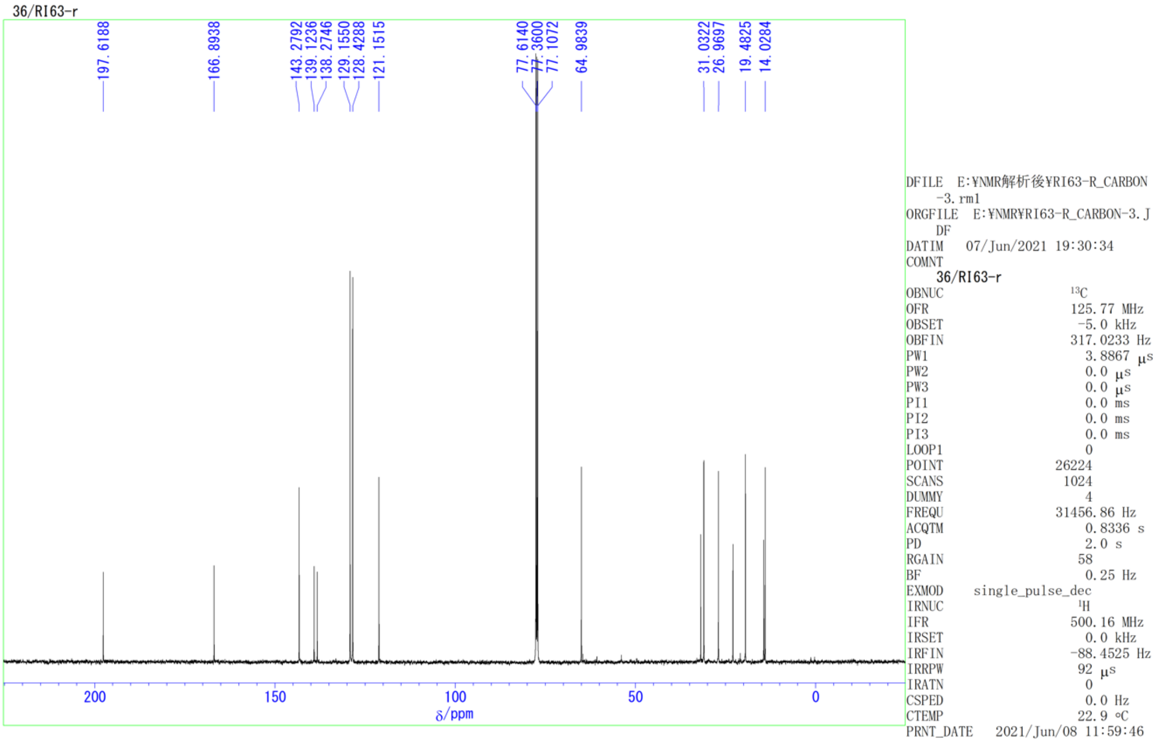

2.12. Butyl (E)-3-(4-methoxyphenyl)acrylate ***5da***.

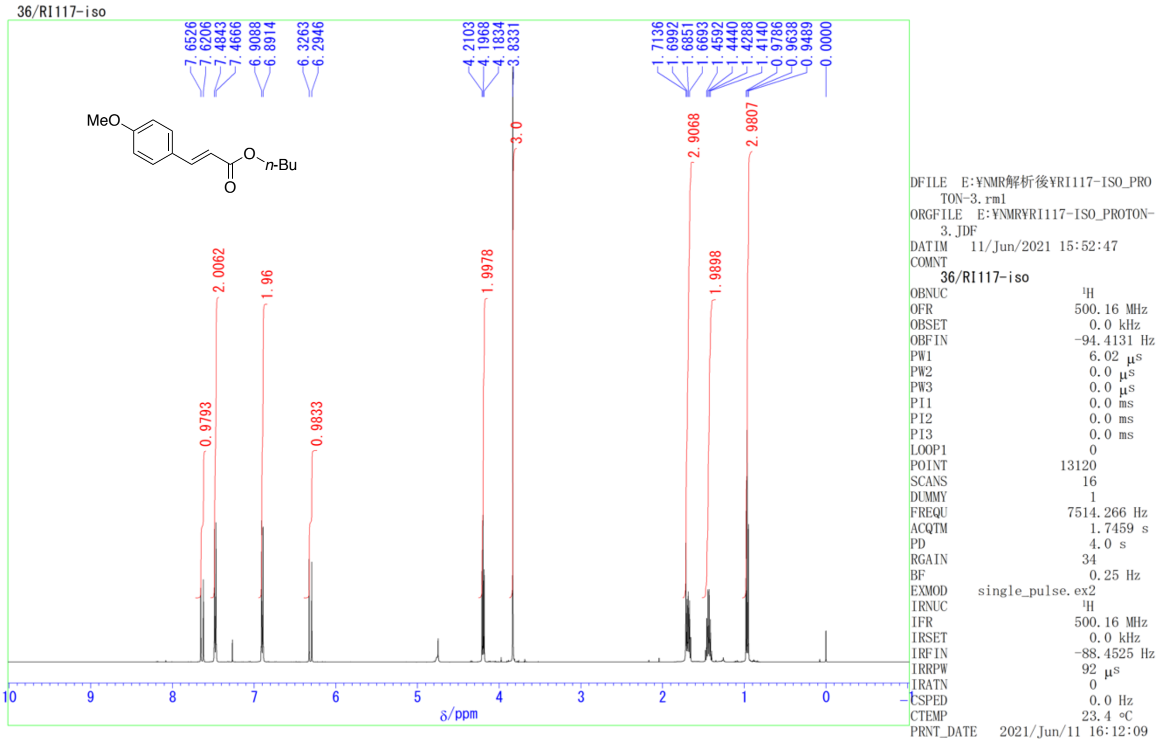

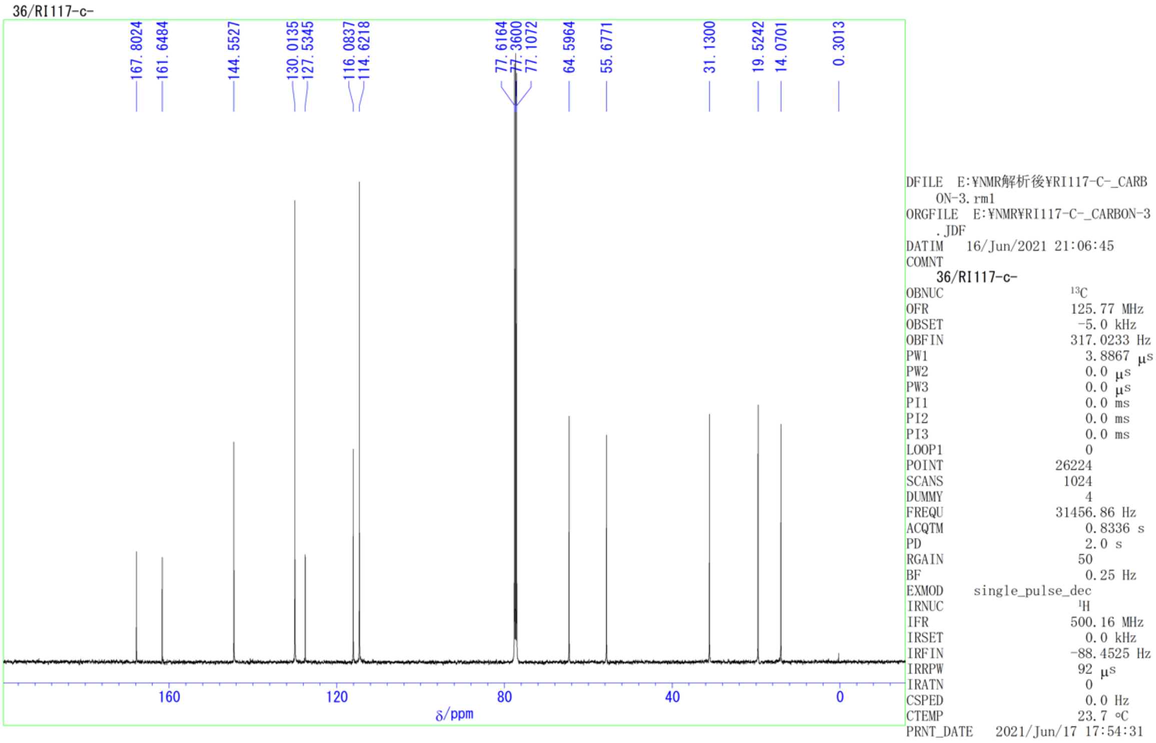

2.13. Butyl (E)-3-(p-tolyl)acrylate ***5ea***.

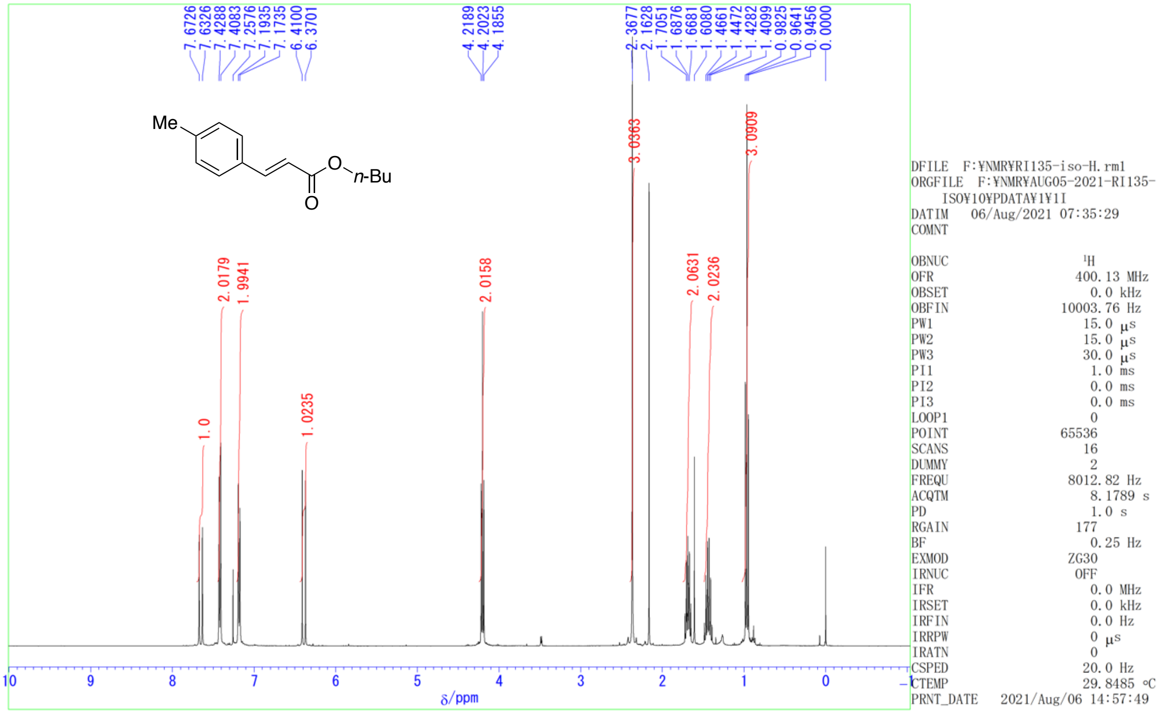

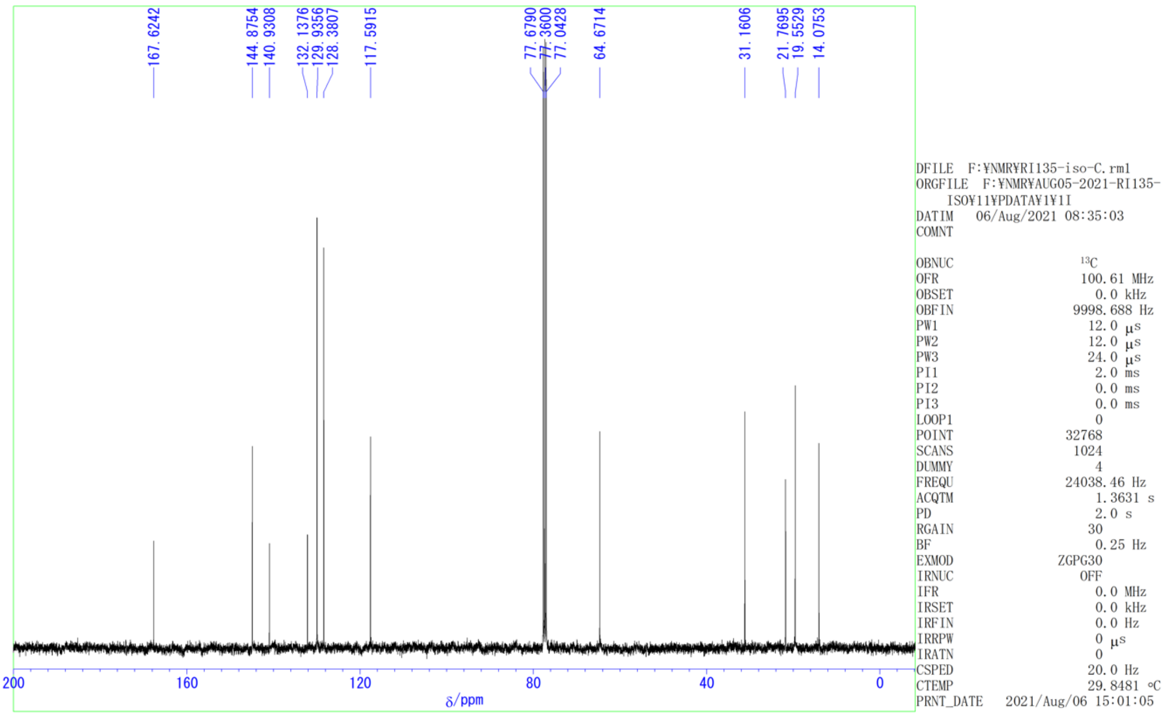

2.14. *Butyl (E)-3-(4-nitrophenyl)acrylate* ***5fa***.

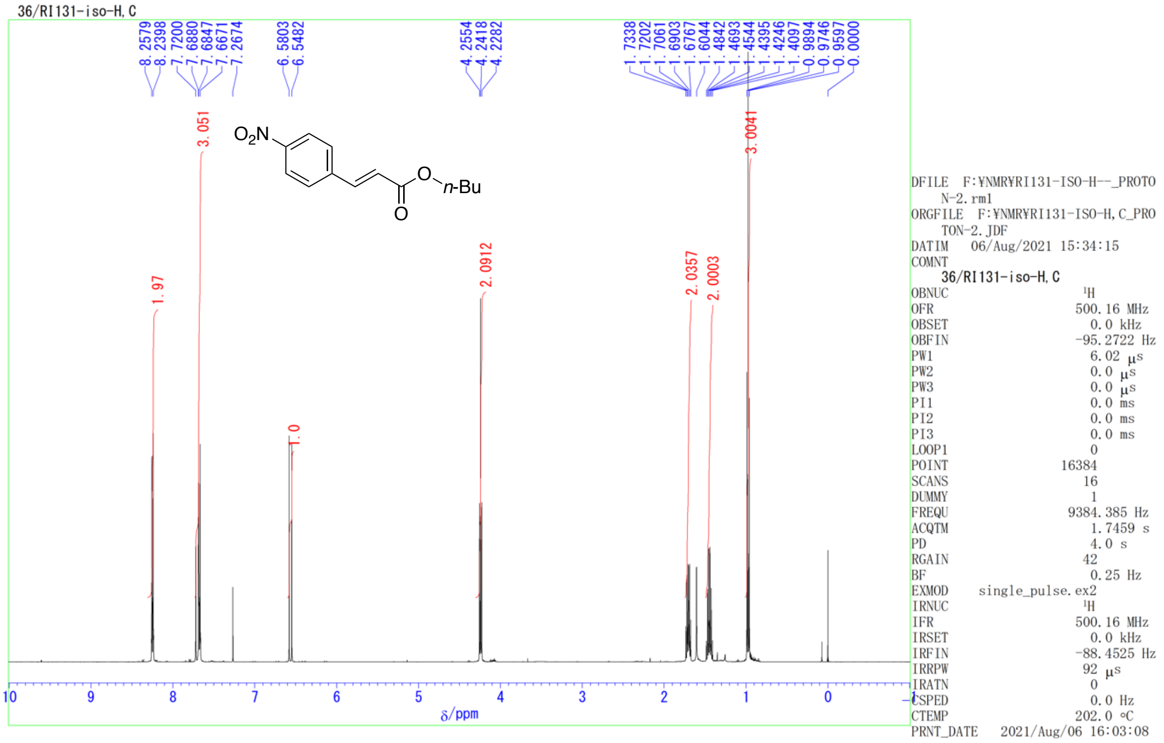

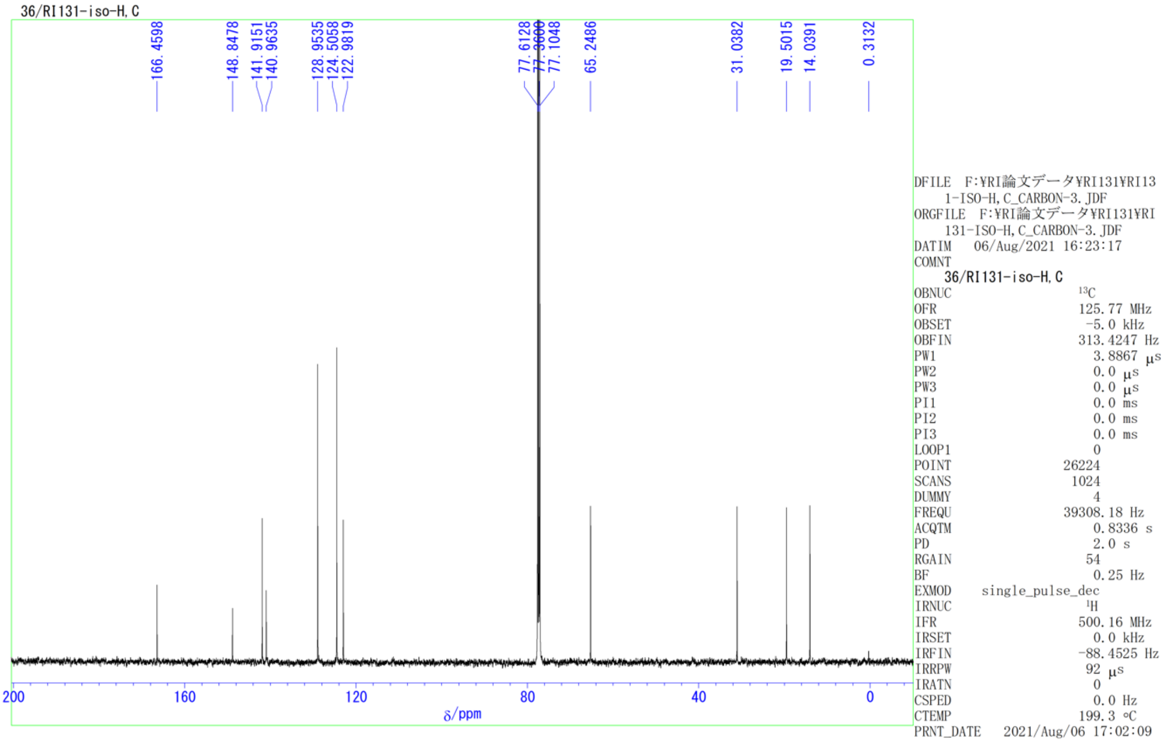

2.15. Butyl (E)-3-(4-(trifluoromethyl)phenyl)acrylate ***5ga****.*

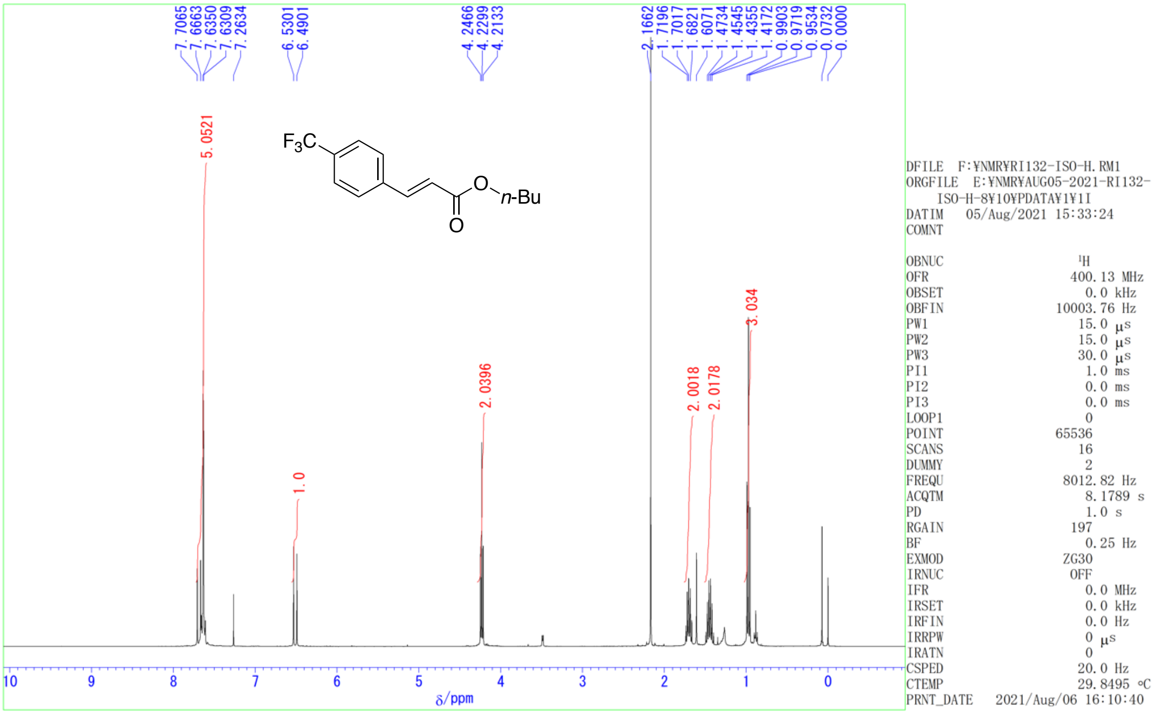

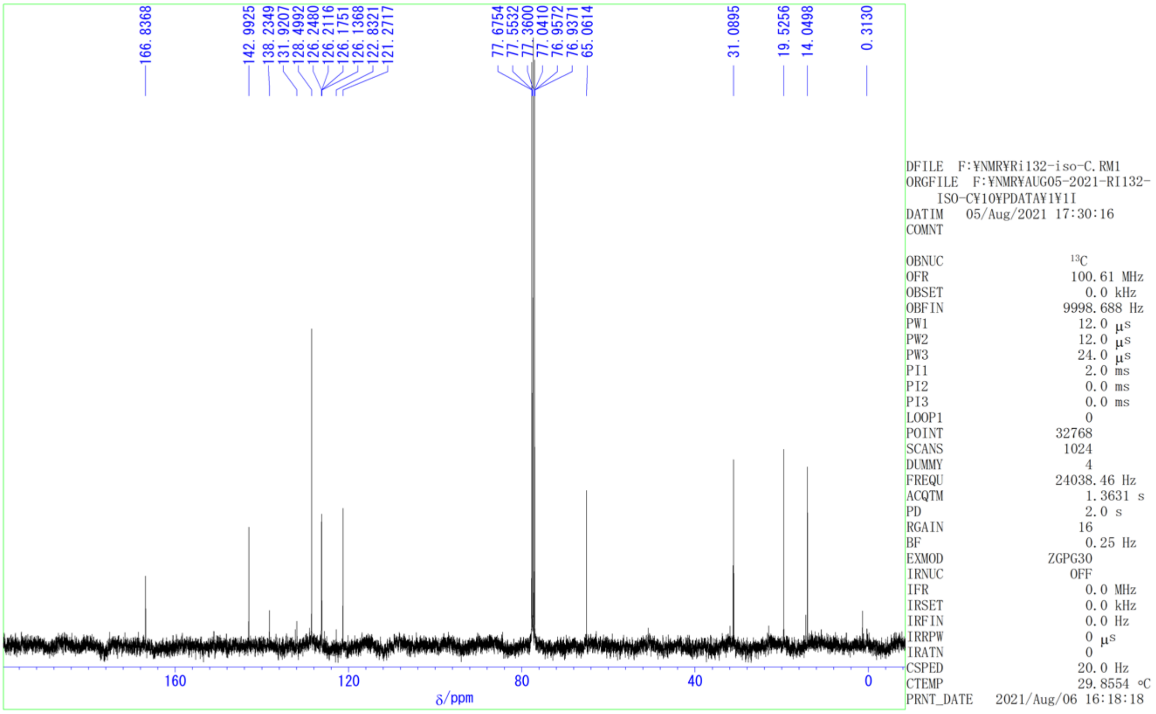

2.16. N-Isopropyl-(E)-cinnamamide ***5ac***.

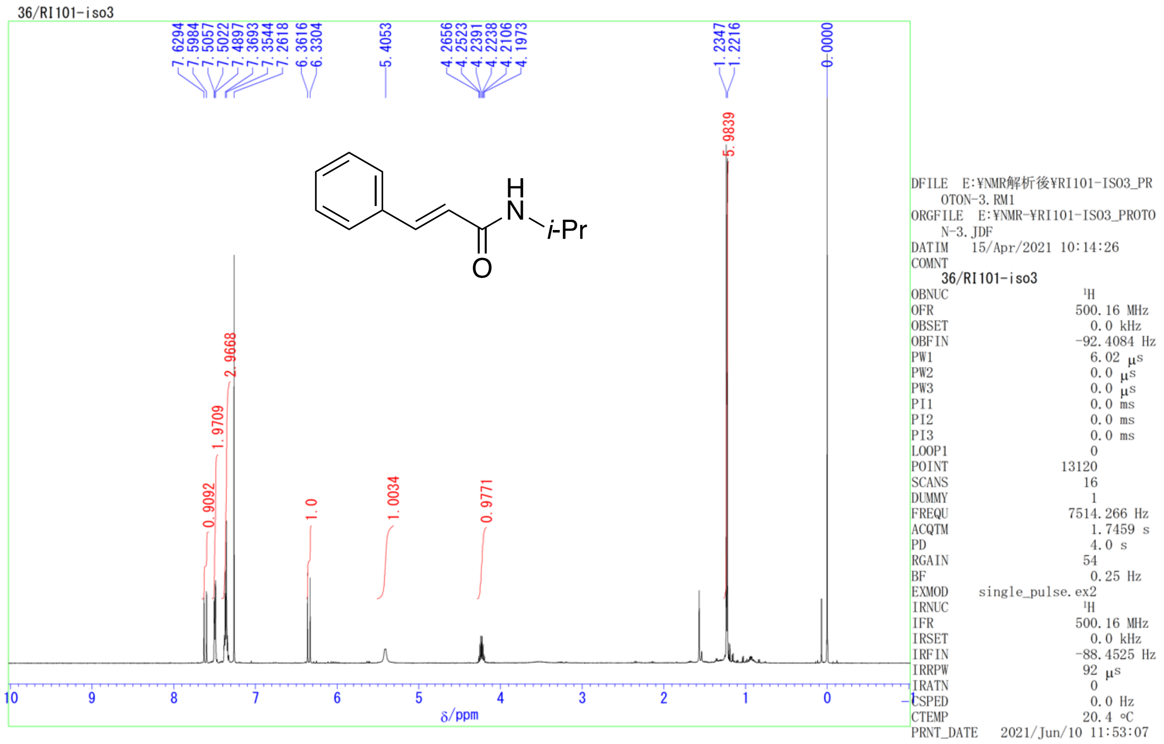

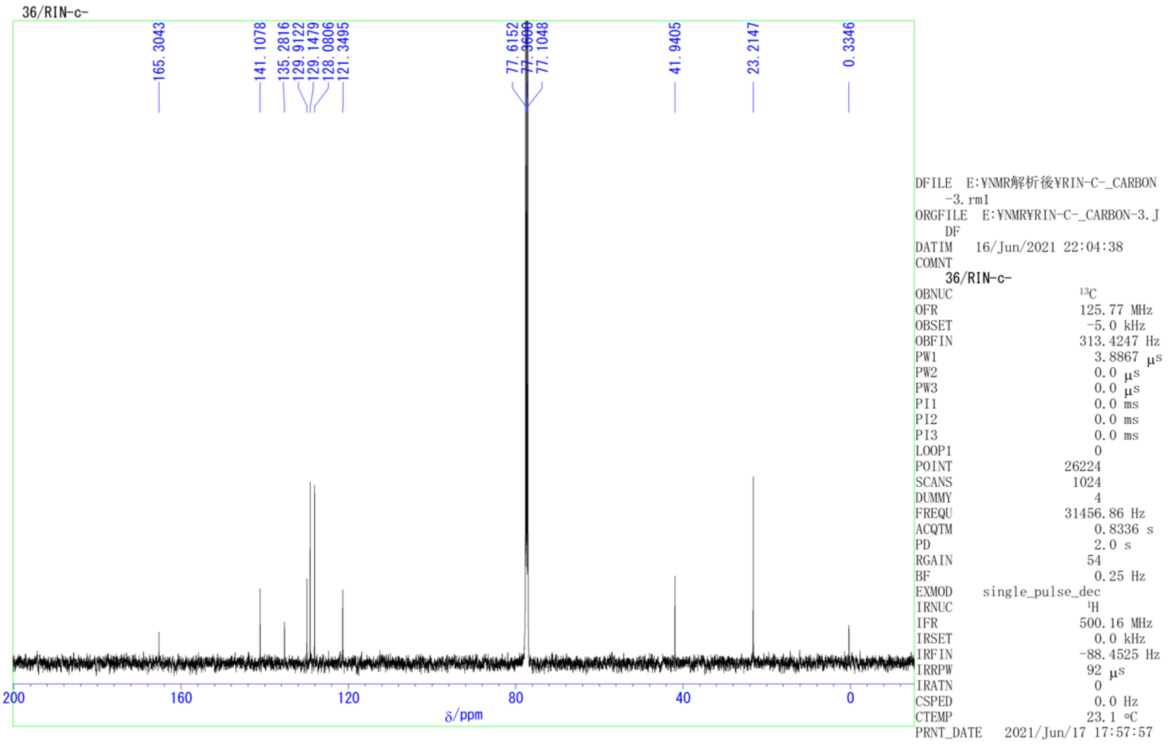

2.17. (E)-Stilbene ***5ad****.*

Hexane (solvent) is contained.

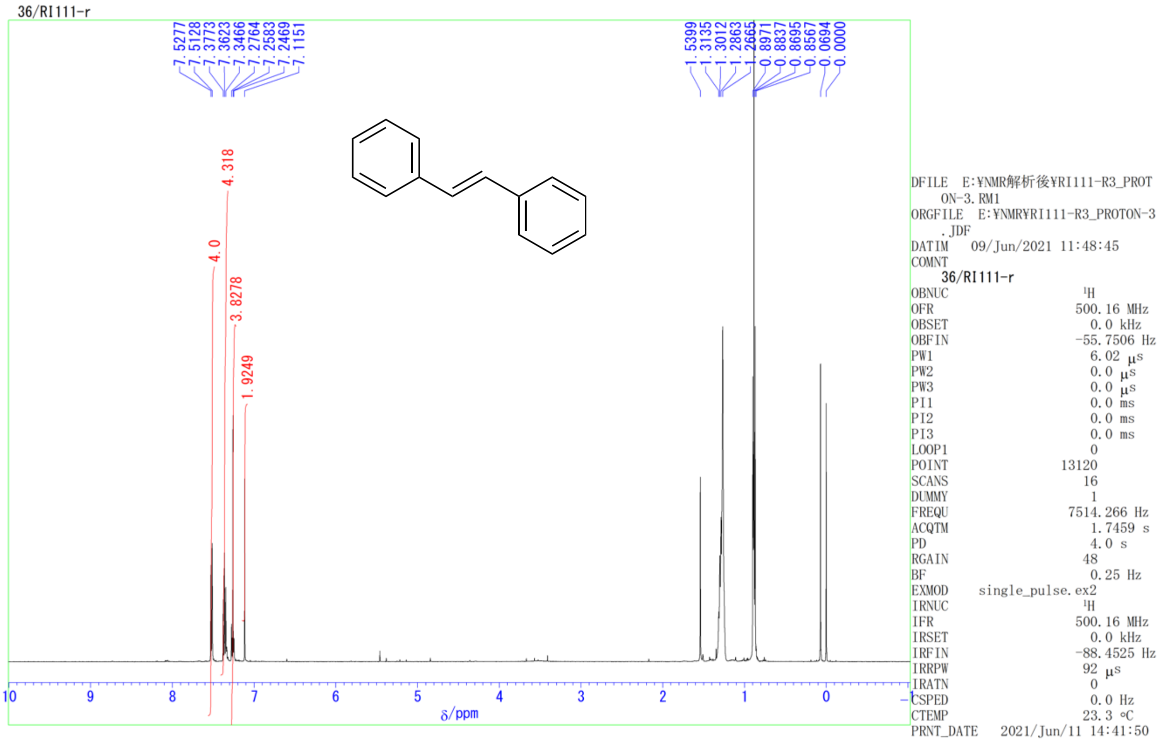

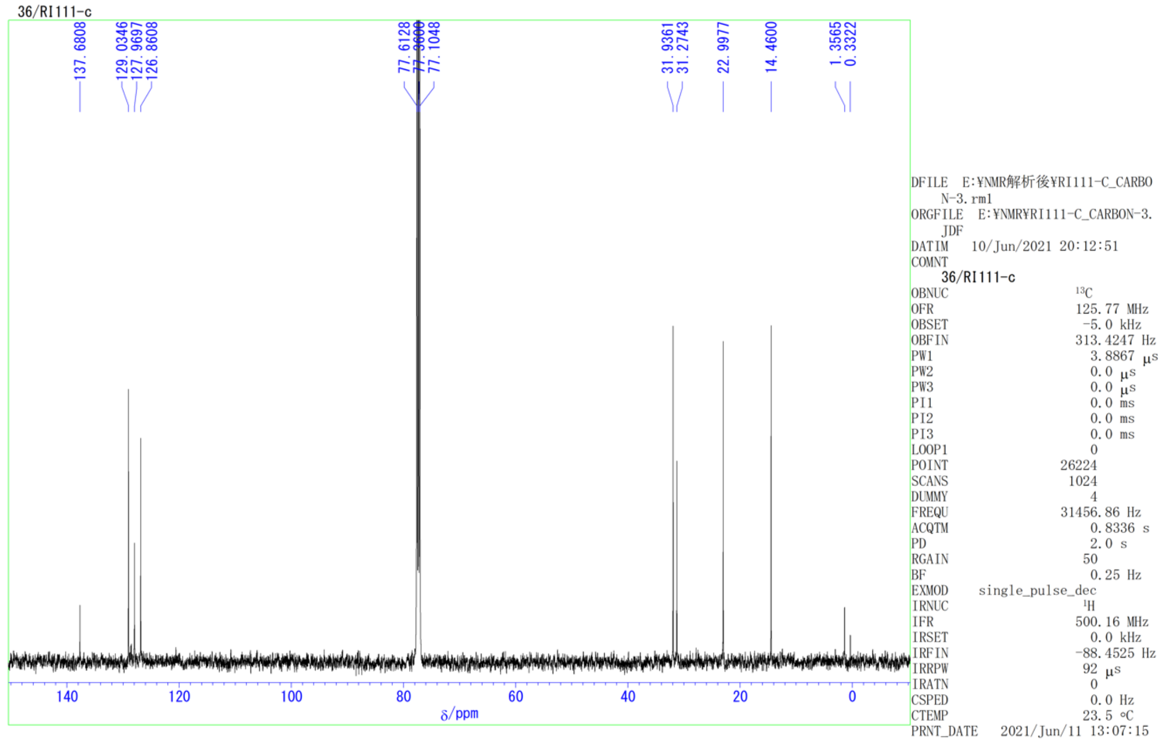

2.18. *(E)-1-Methyl-2-styrylbenzene **5ae***.

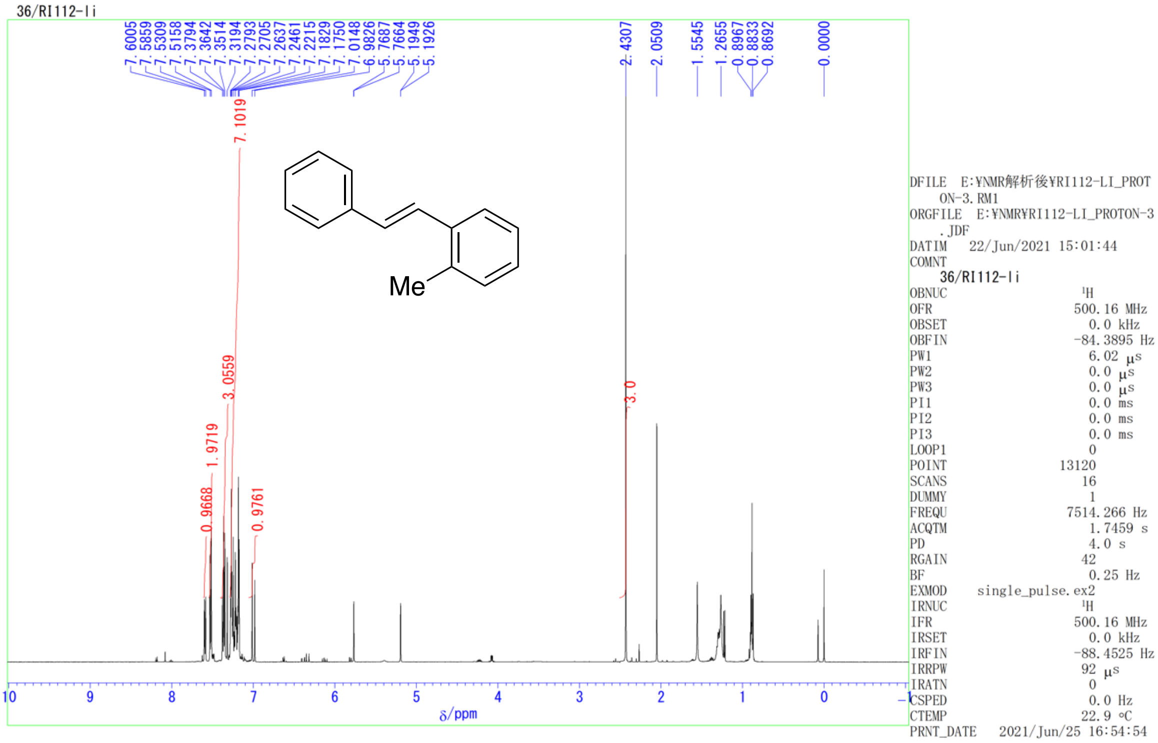

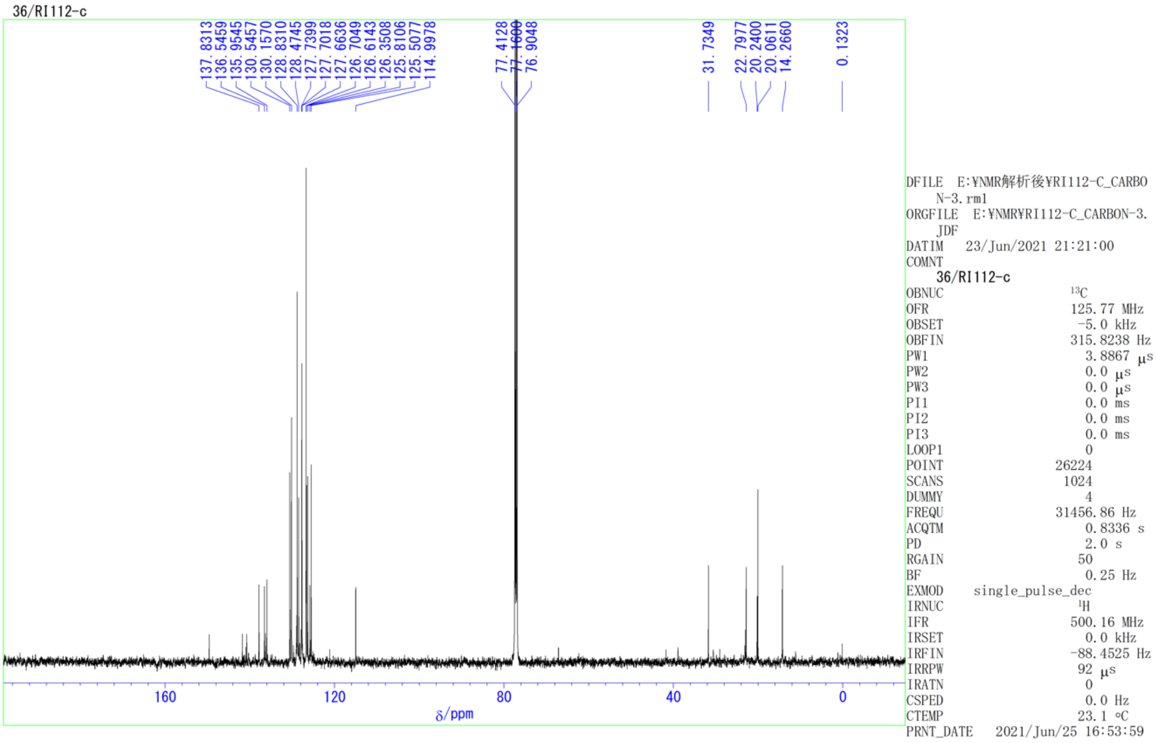

2.19. (E)-1-fluoro-4-styrylbenzene ***5af****.*

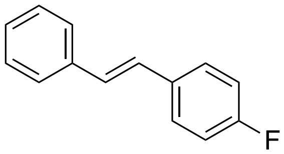

The ^{1}H NMR spectroscopy of the reaction solution was measured because o the low yield (4%).

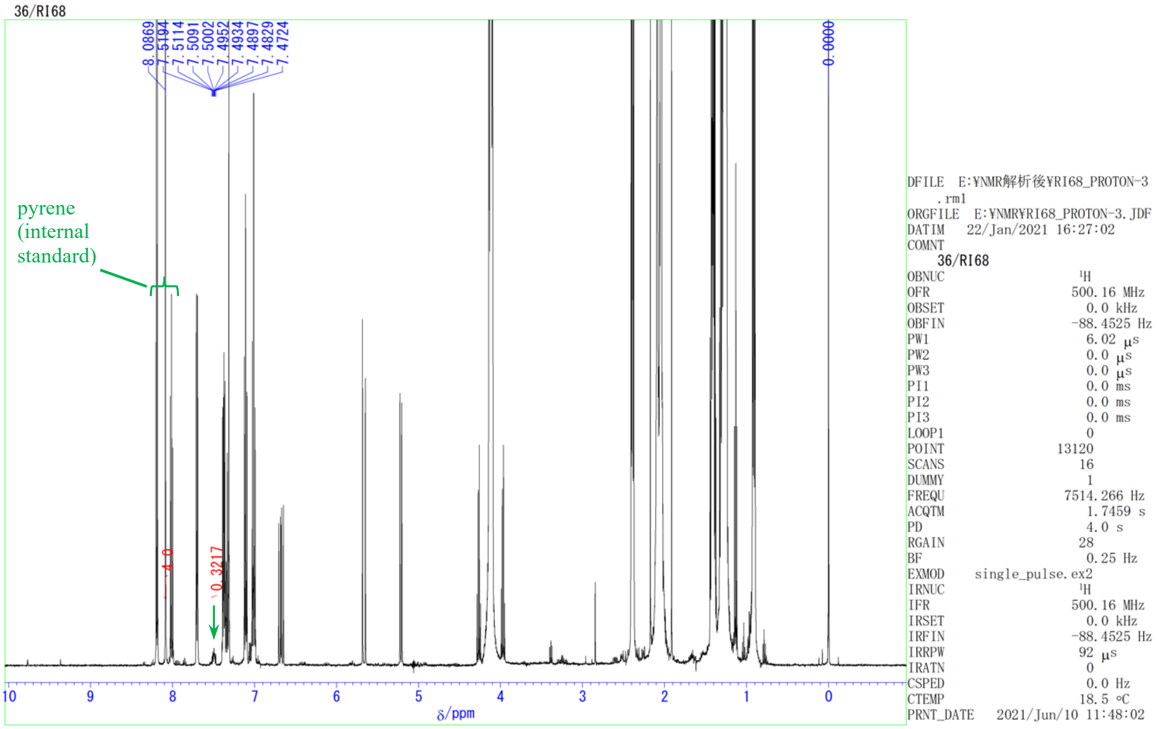

2.20. Butyl (E)-3-(thiophen-2-yl)acrylate ***5ja****.*

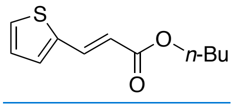

The ^{1}H NMR of the reaction solution; the formation of **5ja** in 6% can be detected (■).

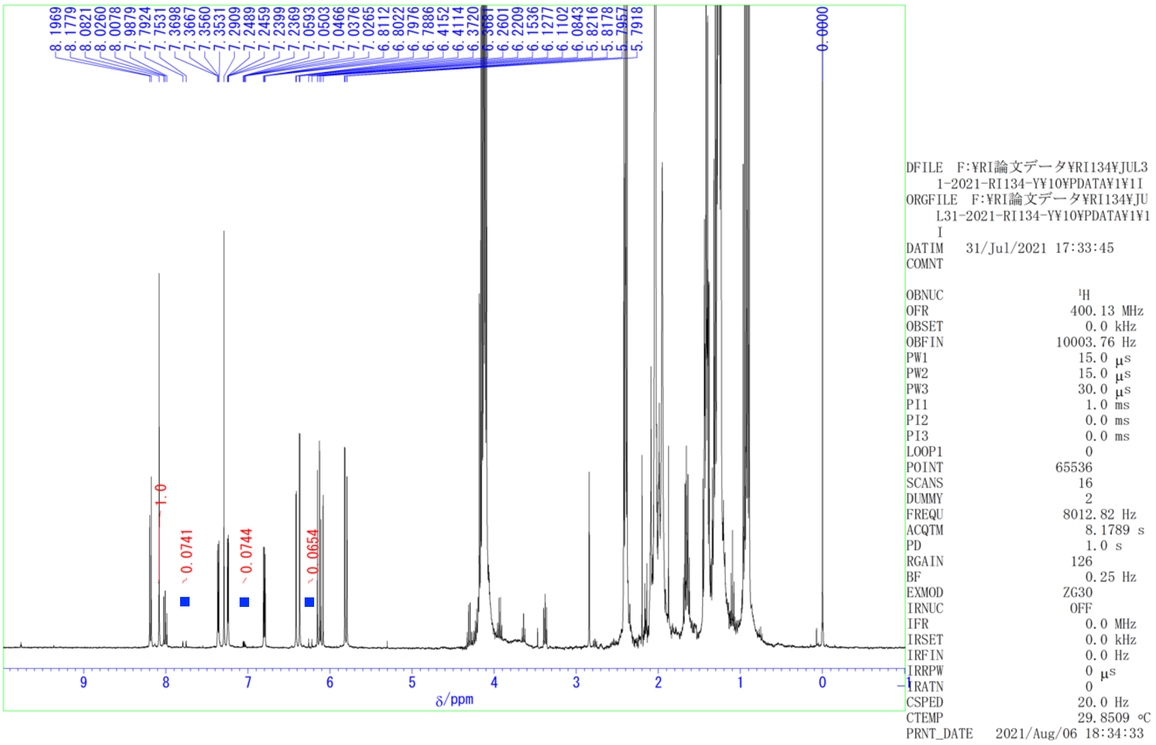

Sonogashira products

*2.21. 1-Methoxy-4-(phenylethynyl)benzene **7da***

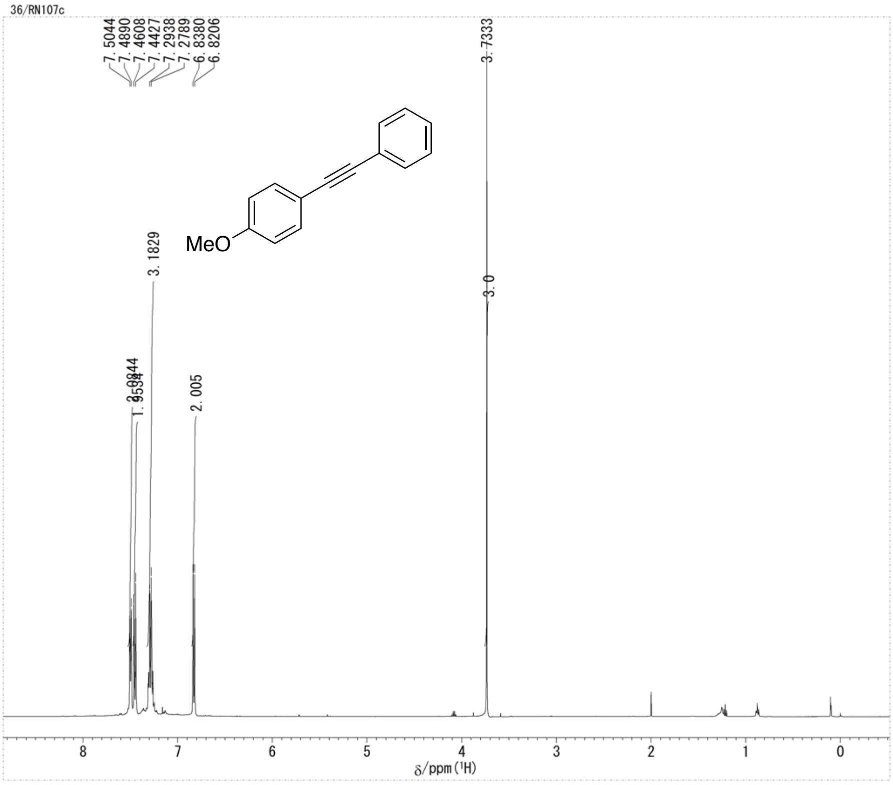

DFILE E:¥RN107C_PROTON-3.JDF
ORGFILE E:¥RN107C_PROTON-3.JDF
DATIM 25/Dec/2020 17:08:10
COMNT
36/RN107c
OBNUC ¹H
OFR 500.16 MHz
OBSET 0.0 kHz
OBFIN -88.4525 Hz
PW1 6.02 μs
PW2 0.0 μs
PW3 0.0 μs
PI1 0.0 ms
PI2 0.0 ms
PI3 0.0 ms
LOOP1 0
POINT 13120
SCANS 16
DUMMY 1
FREQU 9384.385 Hz
ACQTM 1.7459 s
PD 4.0 s
RGAIN 24
BF 0.25 Hz
EXMOD single_pulse.ex2
IRNUC ¹H
IFR 500.16 MHz
IRSET 0.0 kHz
IRFIN -88.4525 Hz
IRRPW 92 μs
IRATN 0
CSPED 0.0 Hz
CTEMP 18.8 °C
PRNT_DATE 2021/Jan/12 18:09:43

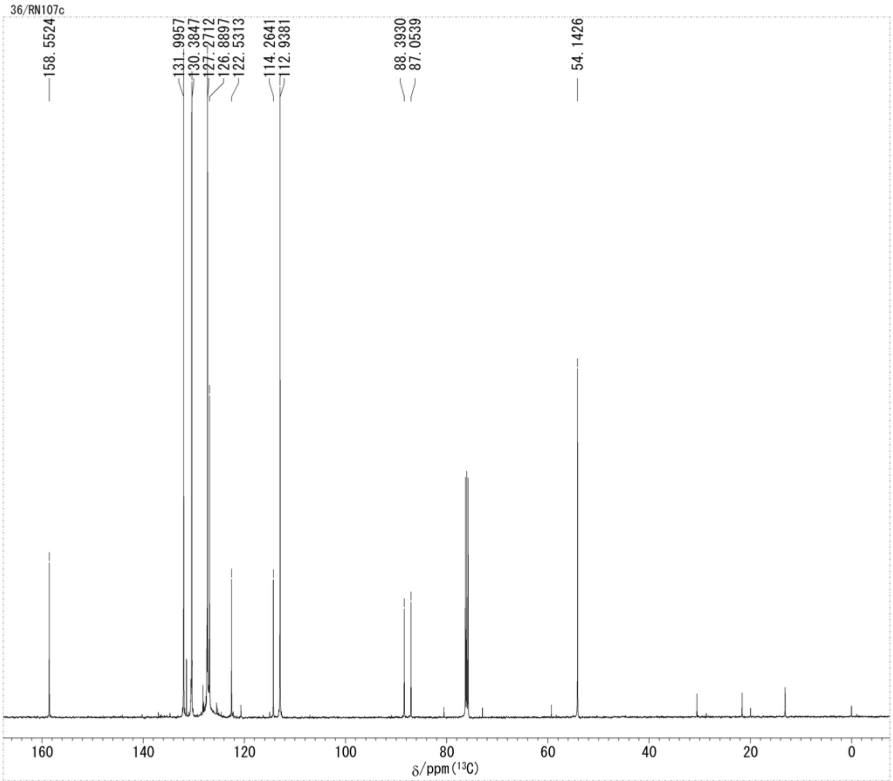

DFILE E:¥RN107C_CARBON-3.JDF
ORGFILE E:¥RN107C_CARBON-3.JDF
DATIM 25/Dec/2020 17:57:07
COMNT
36/RN107c
OBNUC ¹³C
OFR 125.77 MHz
OBSET -5.0 kHz
OBFIN 337.4147 Hz
PW1 3.8867 μs
PW2 0.0 μs
PW3 0.0 μs
PI1 0.0 ms
PI2 0.0 ms
PI3 0.0 ms
LOOP1 0
POINT 26224
SCANS 1024
DUMMY 4
FREQU 39308.18 Hz
ACQTM 0.8336 s
PD 2.0 s
RGAIN 58
BF 0.25 Hz
EXMOD single_pulse_dec
IRNUC ¹H
IFR 500.16 MHz
IRSET 0.0 kHz
IRFIN -88.4525 Hz
IRRPW 92 μs
IRATN 0
CSPED 0.0 Hz
CTEMP 19.5 °C
PRNT_DATE 2021/Jan/12 19:04:20

*2.22. 1-(4-(Phenylethynyl)phenyl)ethan-1-one **7ca**.*

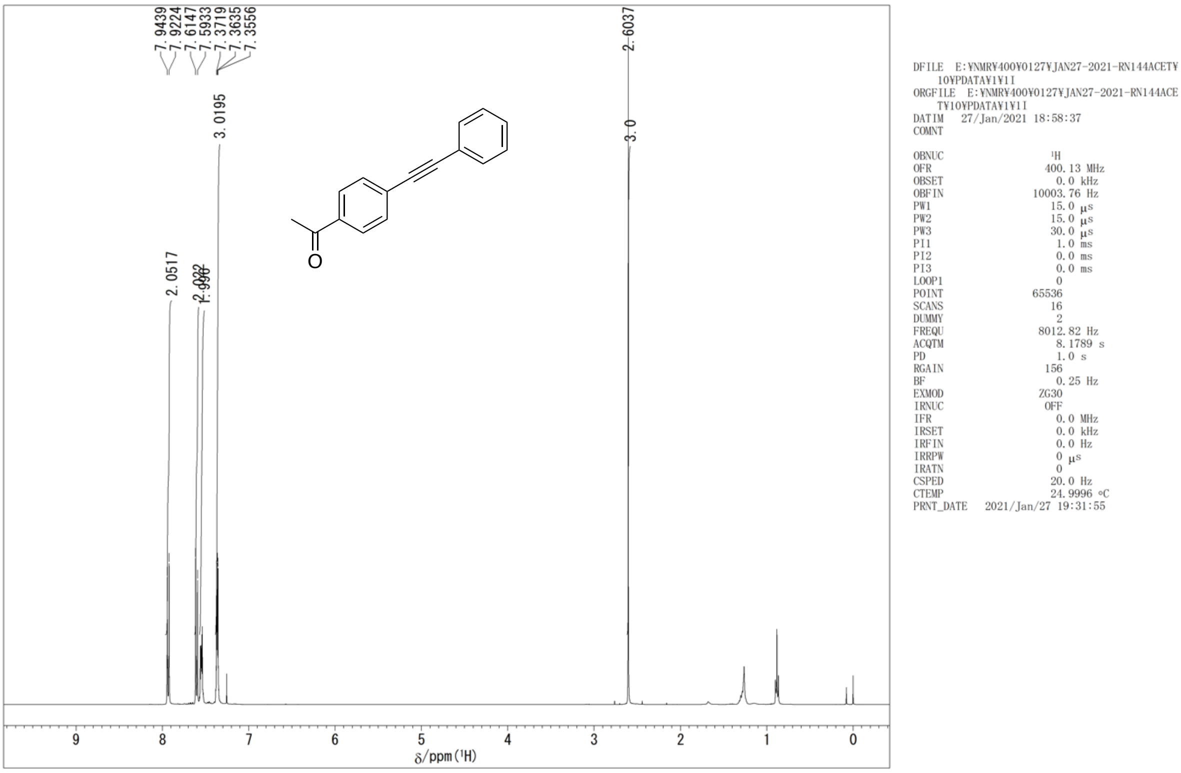

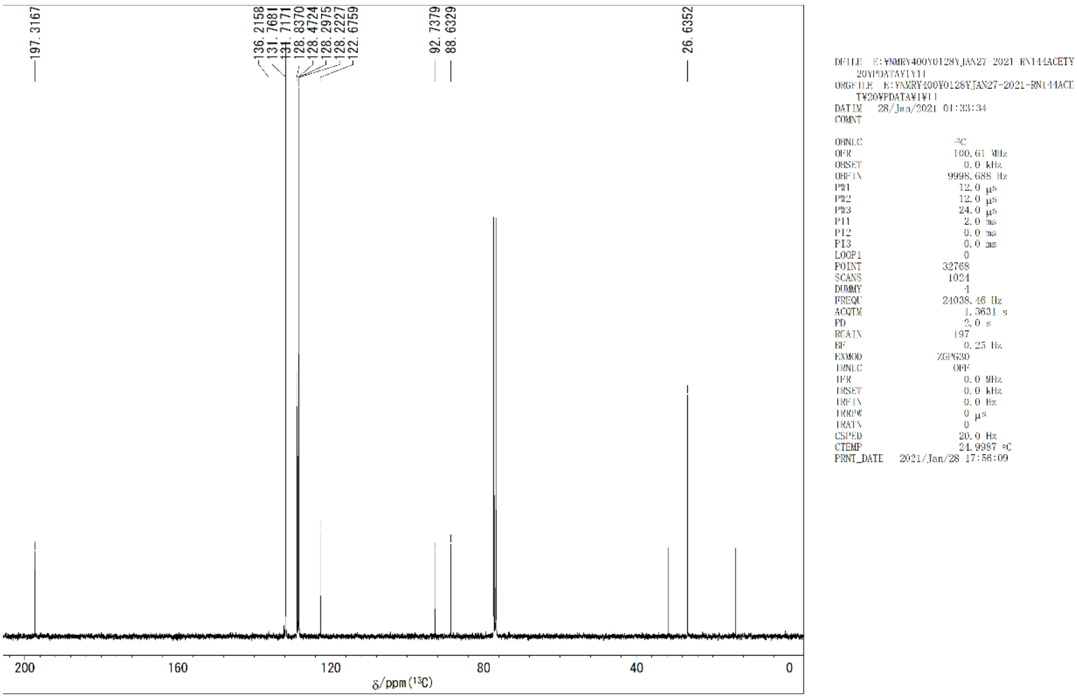

*2.23. 1-(Phenylethynyl)naphthalene **7ka**.*

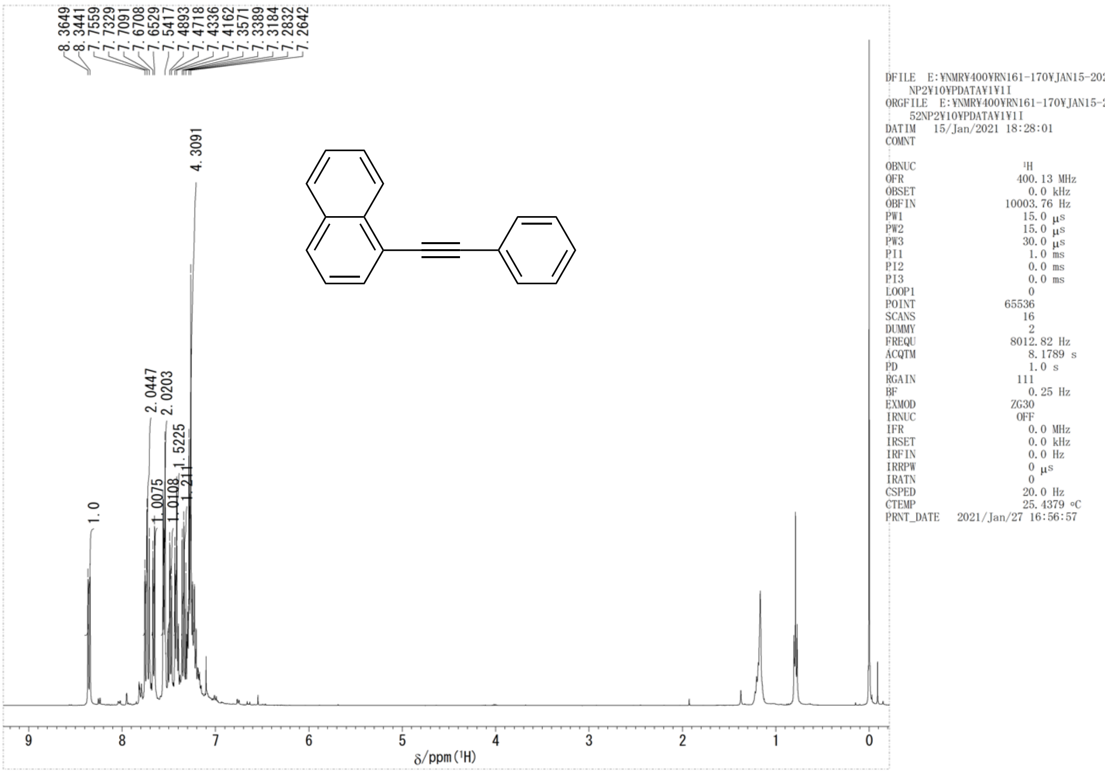

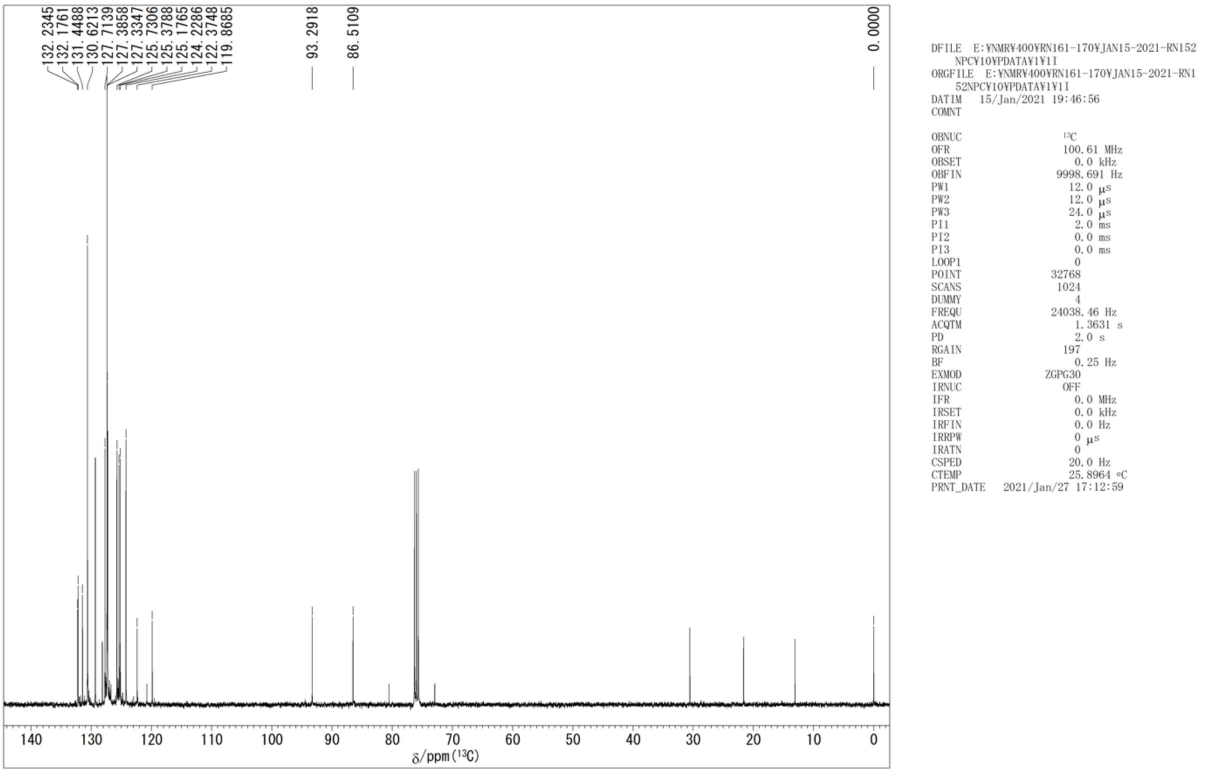

2.24. 2-(Phenylethynyl)thiophene *7ja*

^{1}H NMR of the isolated product, includes small amount of solvents and water.

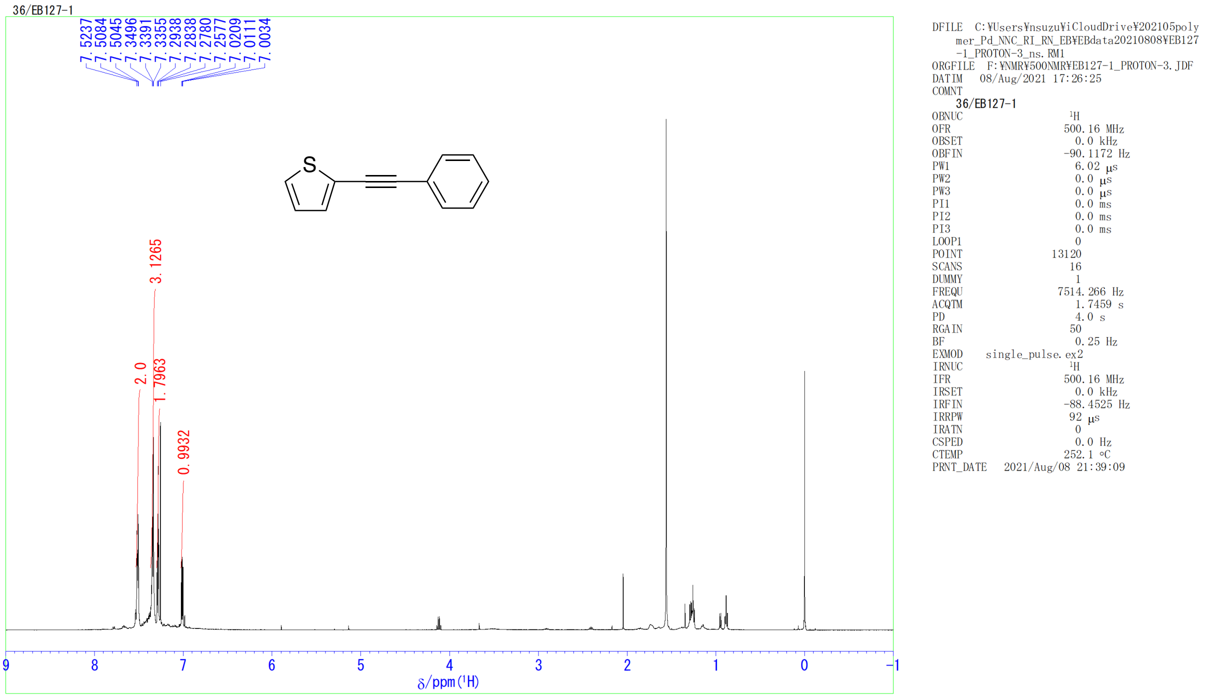

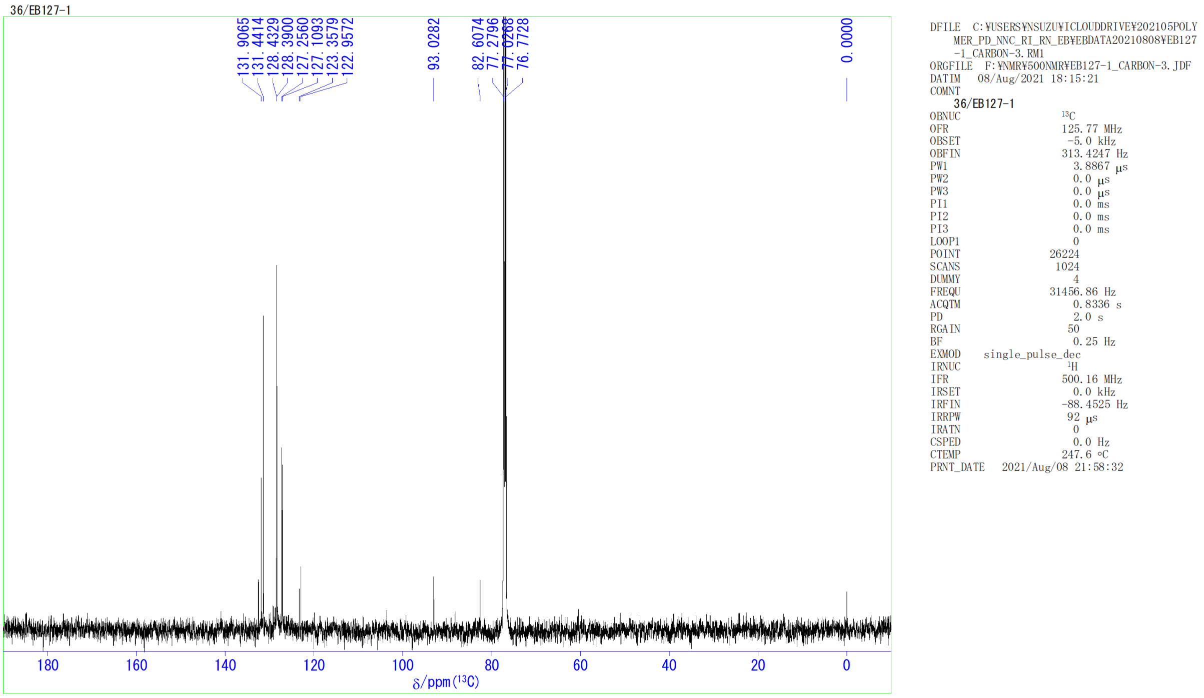

2.25. 4-(Phenylethynyl)benzonitrile **7na**.

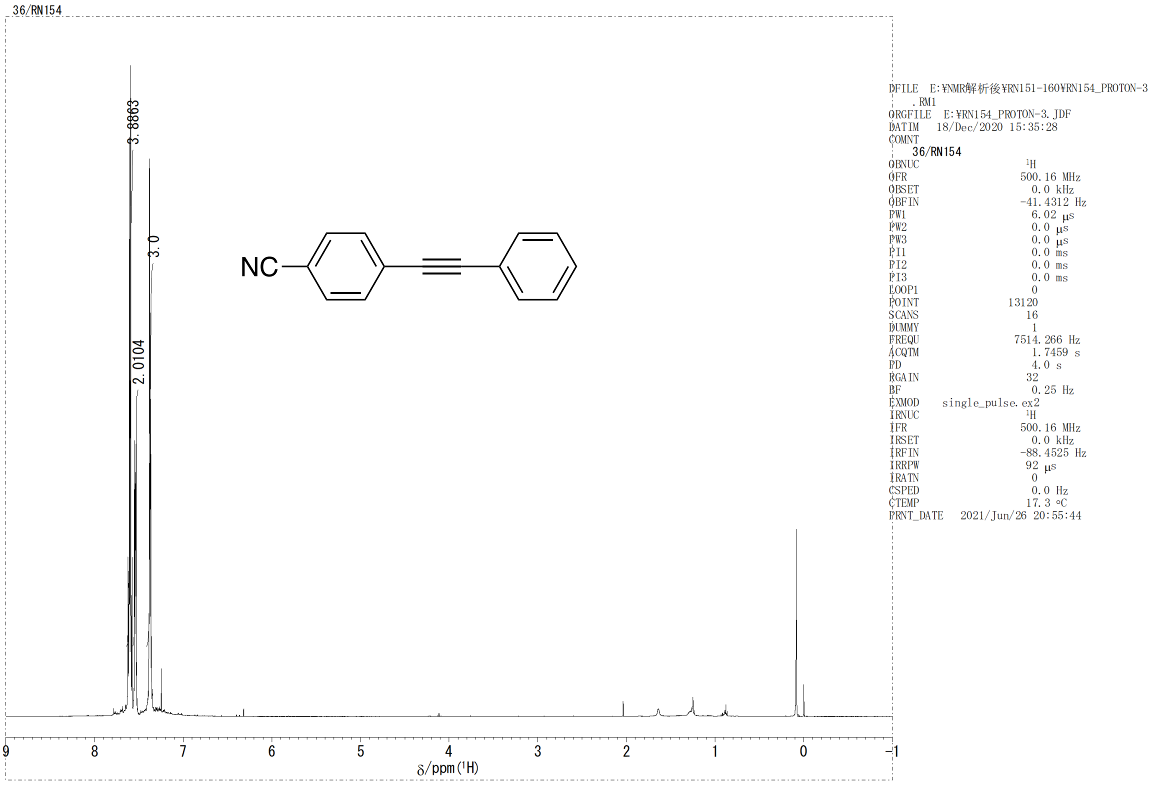

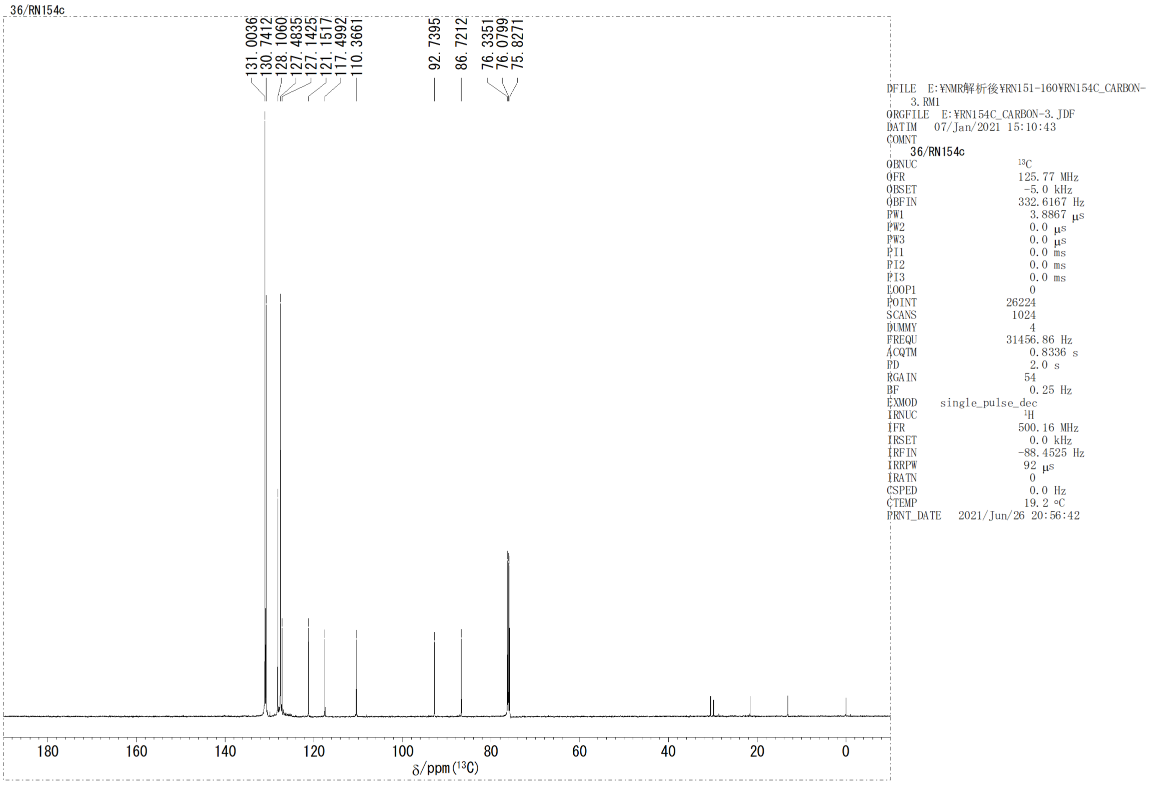

*2.26. 2-(Phenylethynyl)pyridine **7oa**.*

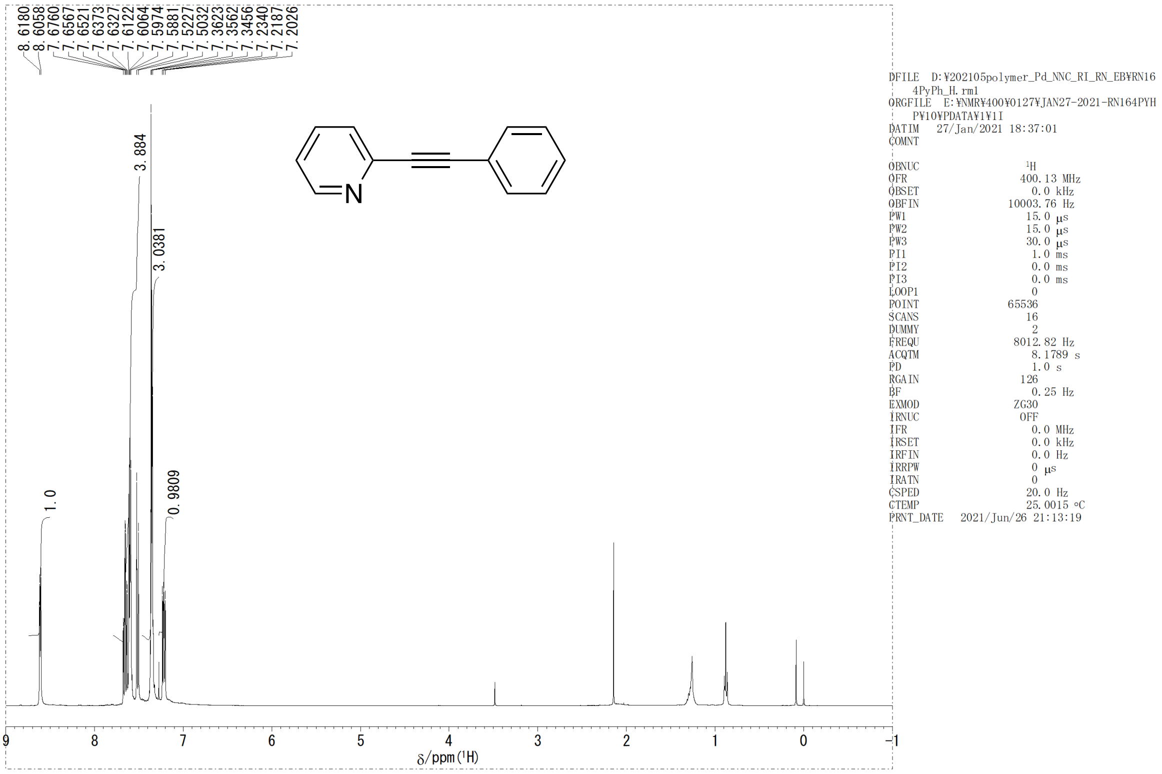

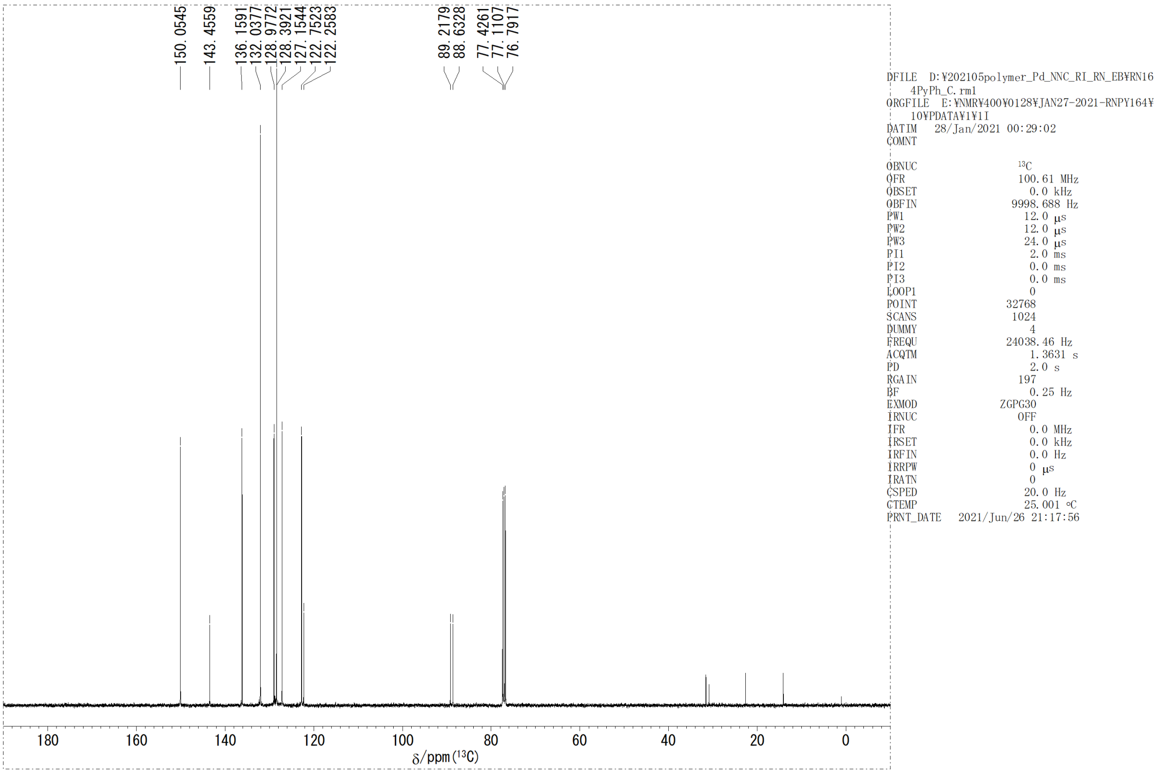

2.27. tert-Butyl((4-methoxyphenyl)ethynyl)diemthylsilane ***7lb***.

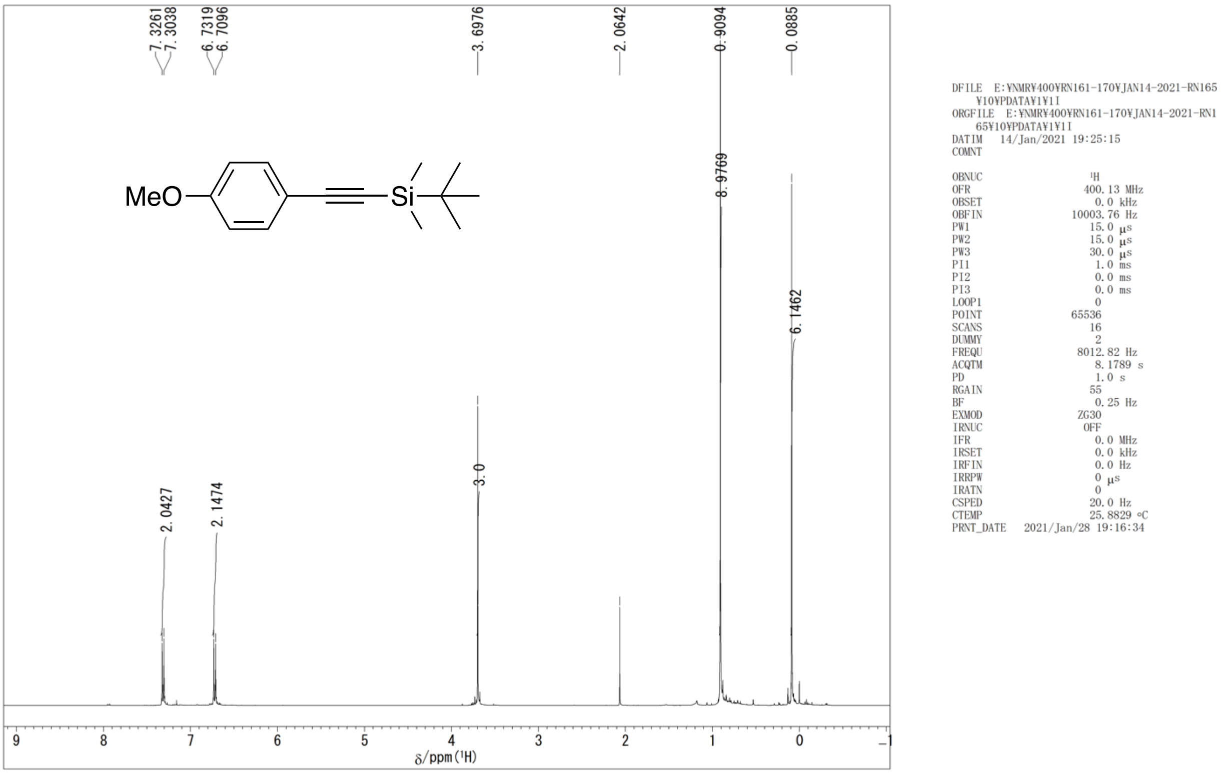

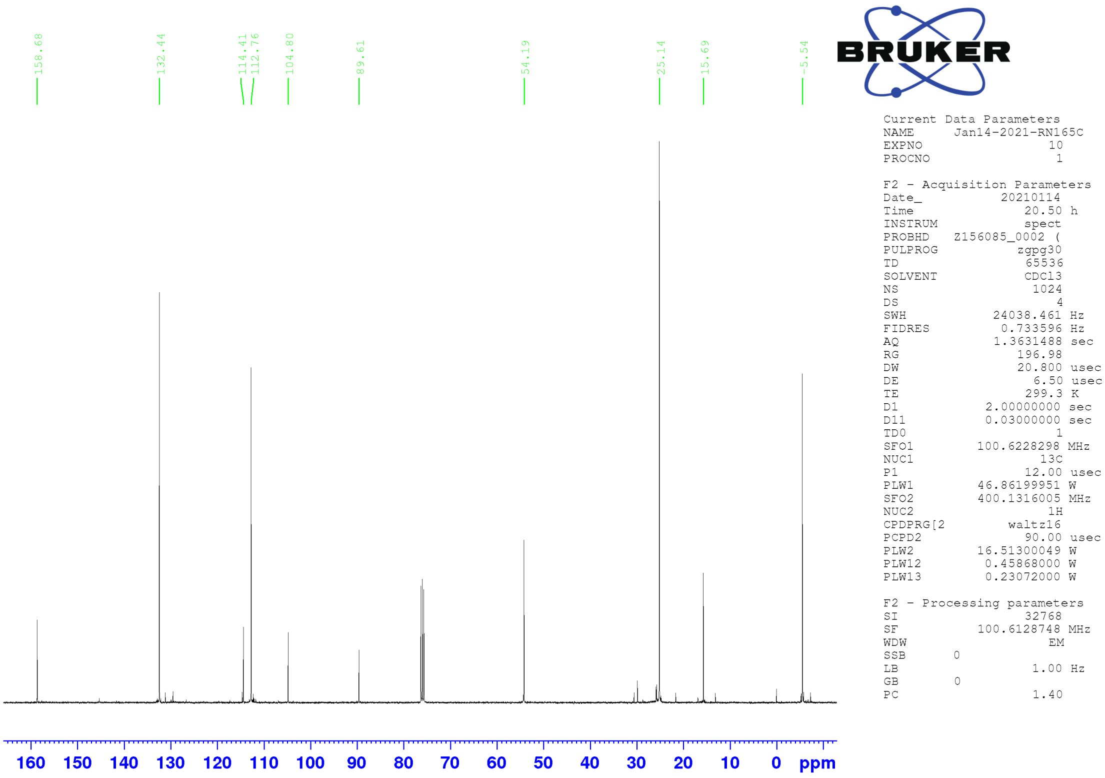

*2.28. 2-((tert-Butyldimethylsilyl)ethynyl)-6-methylpyridine **7pb**.*

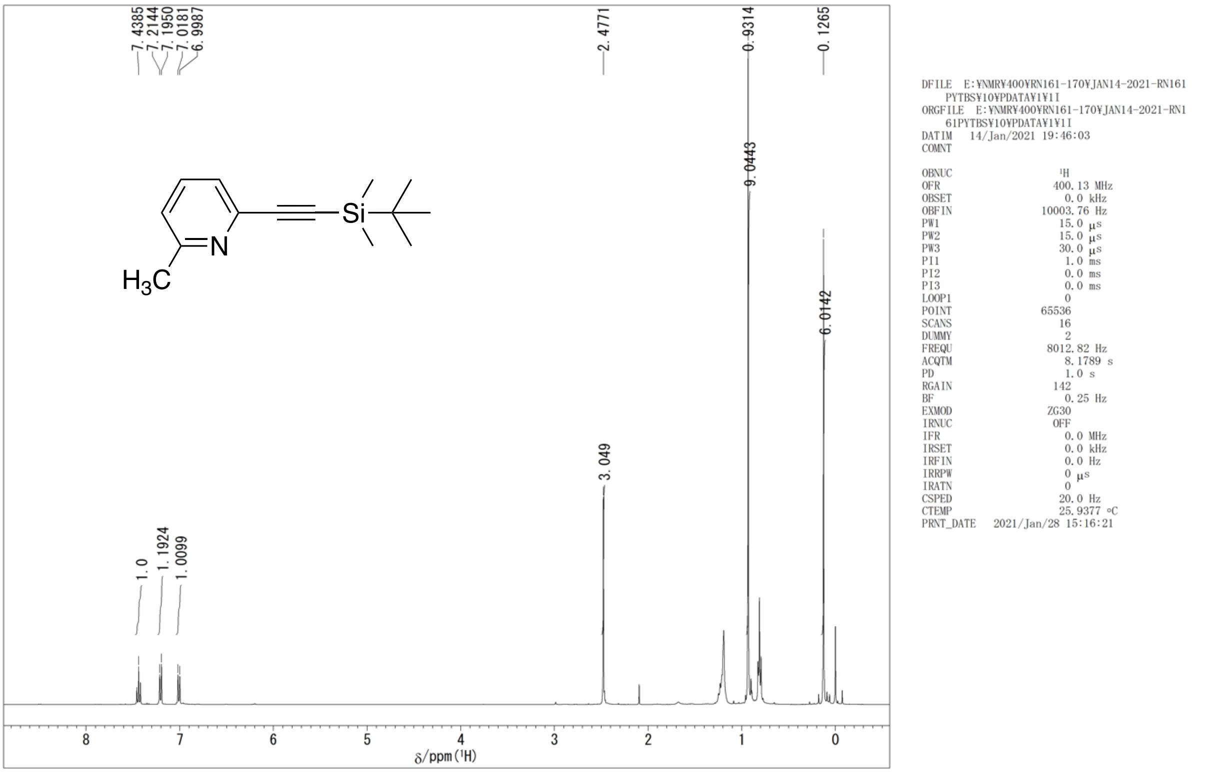

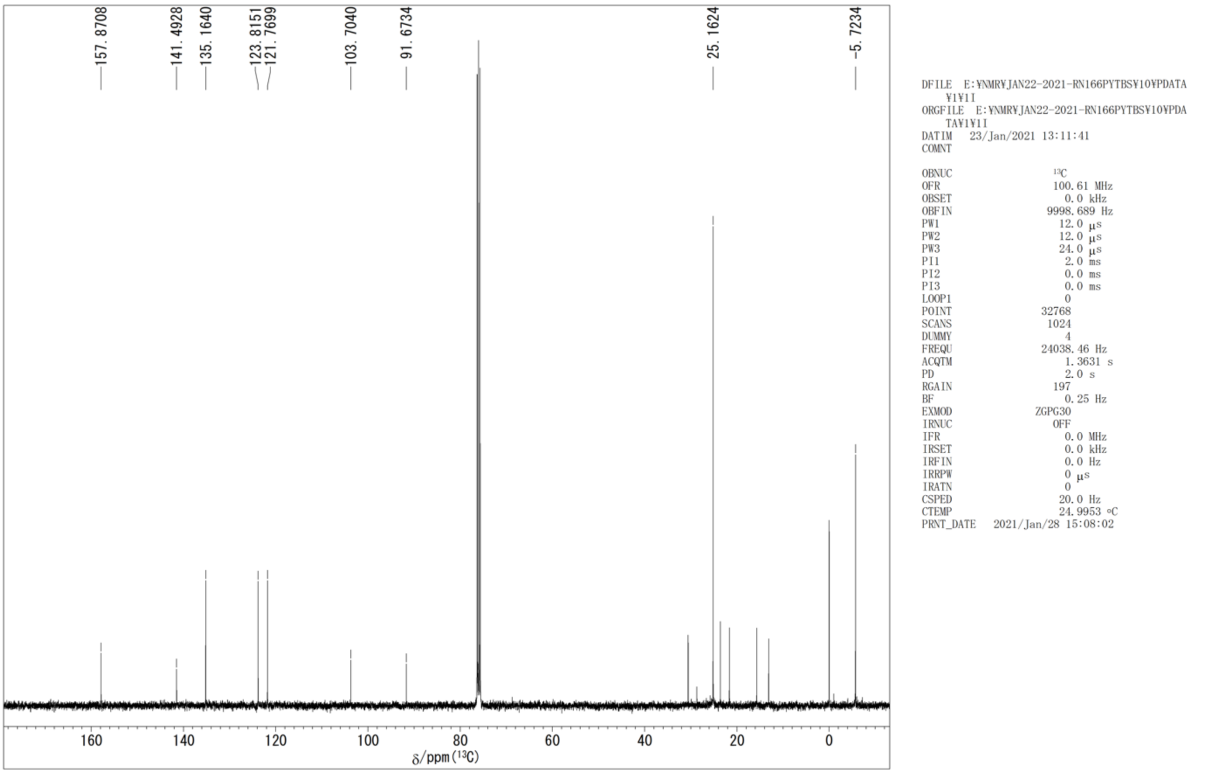

2.29. *1-Methoxy-4-(oct-1-yn-1-yl)benzene **7lc**.*

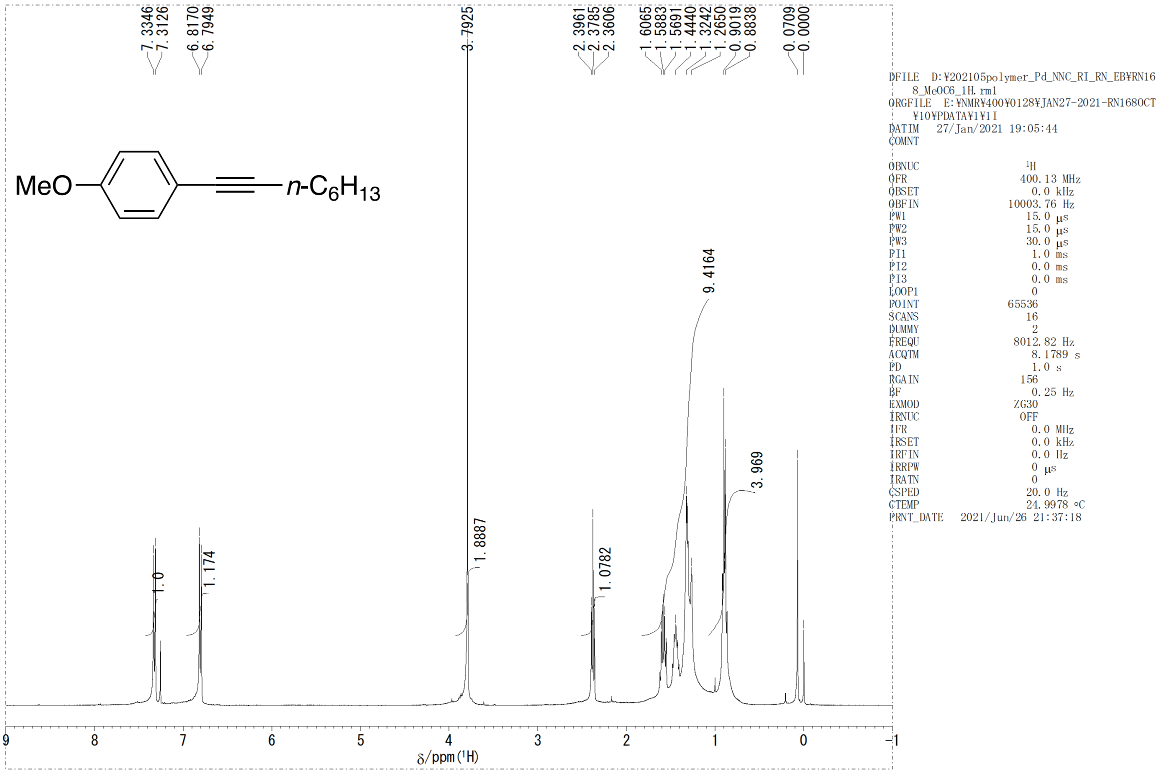

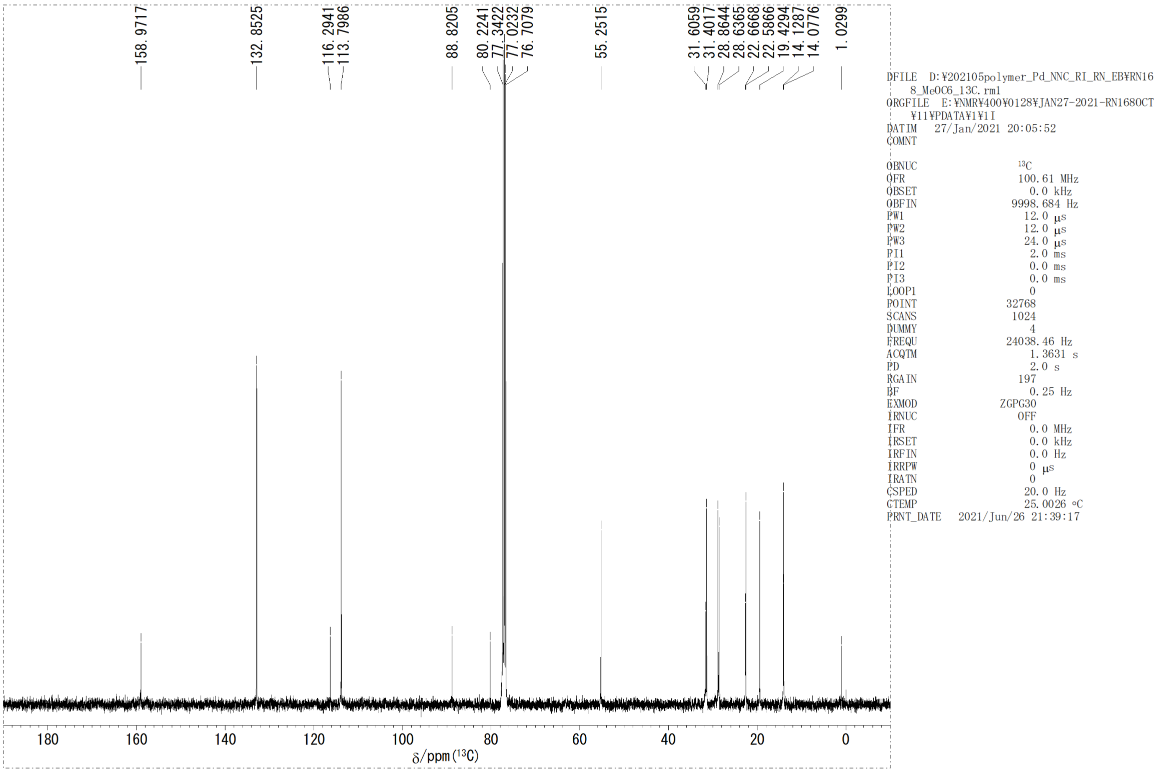

*2.30. (E)-But-1-en-3-yne-1,4-diyldibenzene **7qa**.*

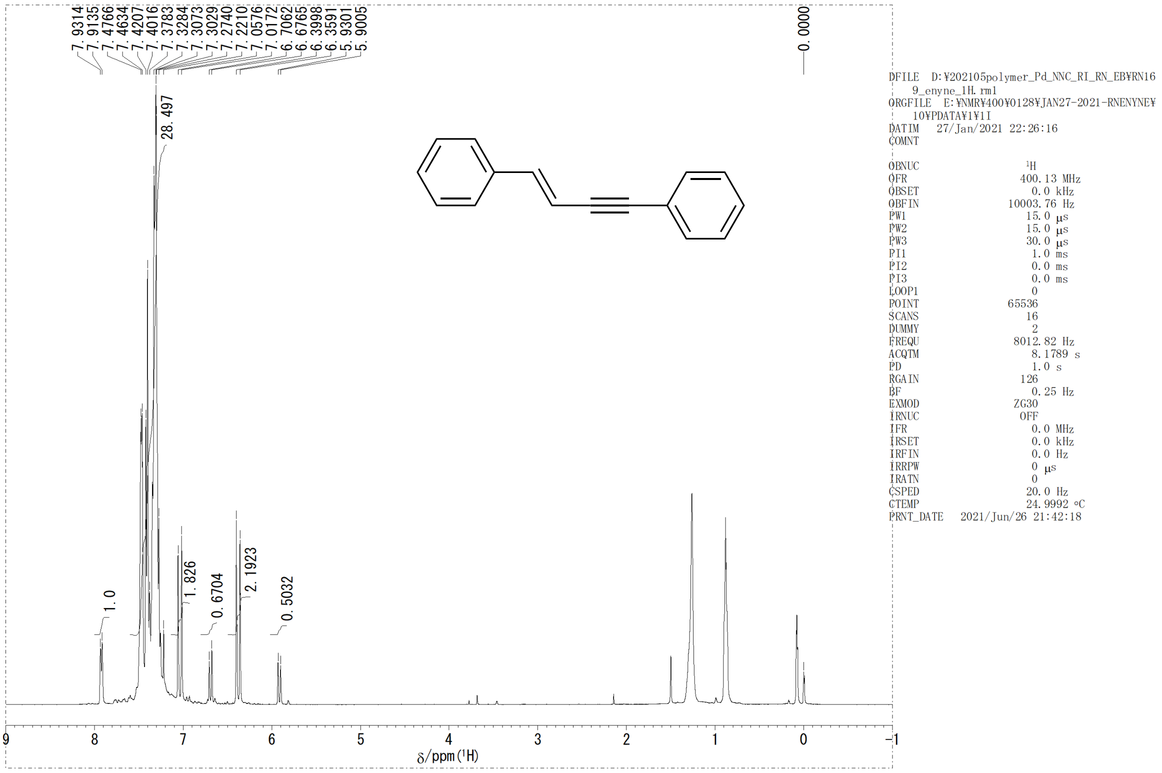

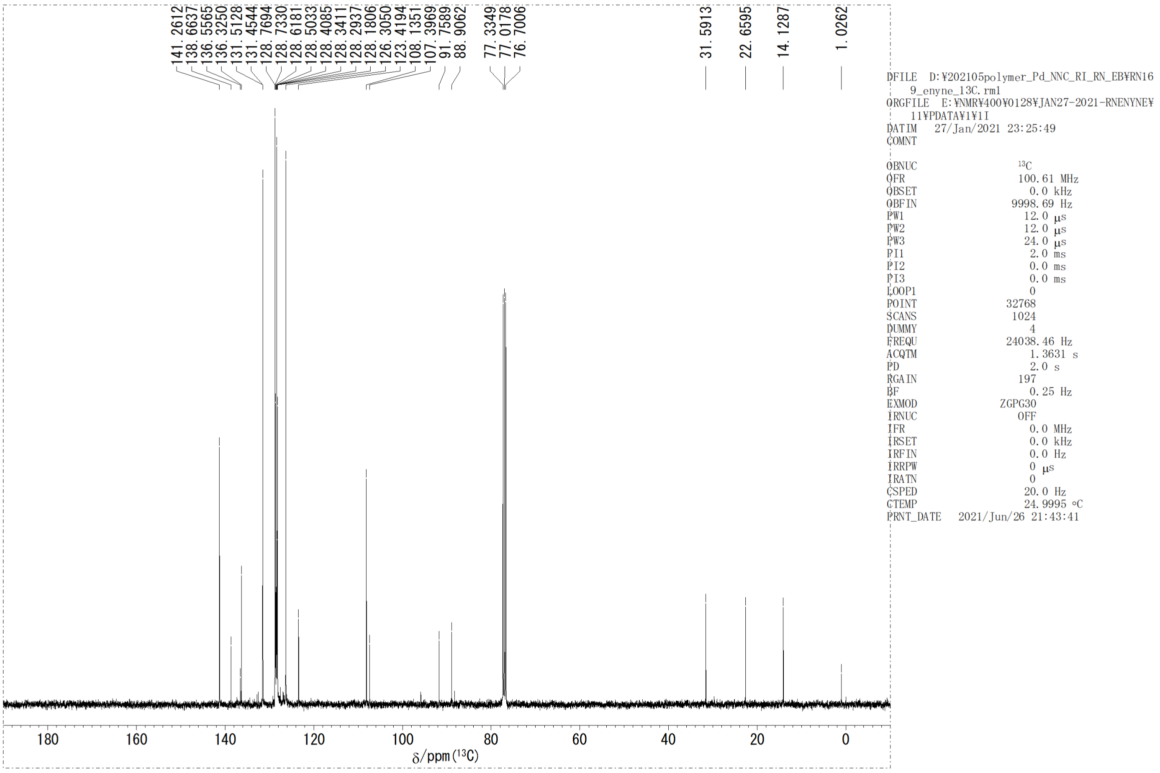

3. References

1. Hamasaka, G.; Ichii, S.; Uozumi, Y., A Palladium NNC-Pincer Complex as an Efficient Catalyst Precursor for the Mizoroki－Heck Reaction. *Adv. Synth. Catal.* **2018,** *360*, 1833-1840.
2. Zehm, D.; Laschewsky, A.; Gradzielski, M.; Prevost, S.; Liang, H.; Rabe, J. P.; Schweins, R.; Gummel, J., Amphiphilic dual brush block copolymers as "giant surfactants" and their aqueous self-assembly. *Langmuir* **2010,** *26*, 3145-55.
3. Flynn, S.; Dale, S. D.; Dwyer, A. B.; Chambon, P.; Rannard, S. P., In situ xanthate deprotection to generate thiol chain transfer agents for conventional free radical linear and branched vinyl polymerization. *J. Polym. Sci. A: Polym. Chem.* **2017,** *55*, 3963-3967.
4. Suzuki, N.; Takabe, T.; Yamauchi, Y.; Koyama, S.; Koike, R.; Rikukawa, M.; Liao, W.-T.; Peng, W.-S.; Tsai, F.-Y., Palladium-catalyzed Mizoroki-Heck reactions in water using thermoresponsive polymer micelles. *Tetrahedron* **2019,** *75*, 1351-1358.
5. Li, J.; Cong, H.; Li, L.; Zheng, S., Thermoresponse improvement of poly(N-isopropylacrylamide) hydrogels via formation of poly(sodium p-styrenesulfonate) nanophases. *ACS Appl. Mater. Interfaces* **2014,** *6*, 13677-87.
6. Kjoniksen, A. L.; Zhu, K.; Pamies, R.; Nystrom, B., Temperature-induced formation and contraction of micelle-like aggregates in aqueous solutions of thermoresponsive short-chain copolymers. *J. Phys. Chem.: B* **2008,** *112*, 3294-3299.
7. Kjøniksen, A.-L.; Zhu, K.; Karlsson, G.; Nyström, B., Novel transition behavior in aqueous solutions of a charged thermoresponsive triblock copolymer. *Colloids Surf. A* **2009,** *333*, 32-45.
8. McFaul, C. A.; Alb, A. M.; Drenski, M. F.; Reed, W. F., Simultaneous multiple sample light scattering detection of LCST during copolymer synthesis. *Polymer* **2011,** *52*, 4825–4833.
9. Behrens, M. A.; Kjøniksen, A.-L.; Zhu, K.; Nyström, B.; Pedersen, J. S., Small-Angle X-ray Scattering Study of Charged Triblock Copolymers as a Function of Polymer Concentration, Temperature, and Charge Screening. *Macromolecules* **2011,** *45*, 246–255.
10. Takeoka, H.; Wada, S.; Yusa, S.-i.; Sakurai, S.; Nakamura, Y.; Fujii, S., Thermo-Responsive Polypyrrole-Palladium NanocompositeParticles Synthesized by Aqueous Chemical OxidativeDispersion Polymerization. *J. Adhes. Soc. Jpn.* **2015,** *51*, 255–263.
11. Mizusaki, M.; Endo, T.; Nakahata, R.; Morishima, Y.; Yusa, S.-i., pH-Induced Association and Dissociation of Intermolecular Complexes Formed by Hydrogen Bonding between Diblock Copolymers. *Polymers* **2017,** *9*, 367–368.
12. Farnham, W. B.; Moad, G.; Thang, S. H.; Rizzardo, E.; Fryd, M. Method for removing sulfur-containing end groups. WO2005113612A1, 2004,(CSIRO).
